# *SignalingProfiler* 2.0 a network-based approach to bridge multi-omics data to phenotypic hallmarks

**DOI:** 10.1038/s41540-024-00417-6

**Published:** 2024-08-23

**Authors:** Veronica Venafra, Francesca Sacco, Livia Perfetto

**Affiliations:** 1 Ph.D. Program in Cellular and Molecular Biology, Department of Biology, University of Rome ‘Tor Vergata’, Rome, Italy; 2 Department of Biology, University of Rome ‘Tor Vergata’, Rome, Italy; 3 Department of Biology and Biotechnologies ‘C.Darwin’, University of Rome ‘La Sapienza’, Rome, Italy

**Keywords:** Signal processing, Reverse engineering

## Abstract

Unraveling how cellular signaling is remodeled upon perturbation is crucial for understanding disease mechanisms and identifying potential drug targets. In this pursuit, computational tools generating mechanistic hypotheses from multi-omics data have invaluable potential. Here, we present a newly implemented version (2.0) of *SignalingProfiler*, a multi-step pipeline to draw mechanistic hypotheses on the signaling events impacting cellular phenotypes. *SignalingProfiler* 2.0 derives context-specific signaling networks by integrating proteogenomic data with the prior knowledge-causal network. This is a freely accessible and flexible tool that incorporates statistical, footprint-based, and graph algorithms to accelerate the integration and interpretation of multi-omics data. Through a benchmarking process on three proof-of-concept studies, we demonstrate the tool’s ability to generate hierarchical mechanistic networks recapitulating novel and known perturbed signaling and phenotypic outcomes, in both human and mice contexts. In summary, S*ignalingProfiler* 2.0 addresses the emergent need to derive biologically relevant information from complex multi-omics data by extracting interpretable networks.

## Introduction

Intracellular signaling pathways, marked by molecular interactions and post-translational modifications like phosphorylation, mediate the ability of cells to translate signals into observable changes in phenotypic traits. Numerous pathways (e.g., MAPKs, EGFR, …) have been extensively studied and it is now evident that these linear cascades are not isolated entities, but rather components of a large and complex network that impact physiological and pathological processes^1^. To understand the intricate nature of such a human signaling network it is crucial to grasp the cross-talk among diverse signaling cascades and elucidate how they collectively impact key cellular phenotypes.

The recent tremendous technological advances have enabled the cost-effective generation of large-scale -omics datasets, providing a systematic description of different regulatory layers (e.g., DNA, RNA, and protein levels) in various pathophysiological conditions. The simultaneous exploration of different omics layers in an integrative manner (the so-called, ‘multi-omics data analysis’) is indeed gaining popularity^2–4^ to obtain a holistic picture of the cell state^5^. However, extracting biological information from such complex omics data remains a major challenge and demands computational interventions.

Among the different methods developed^6,7^, footprint-based techniques^8,9^ generate lists of kinases and transcription factors characterized by an activity score derived from the phosphorylation or expression level of their known targets^10–14^. However, how and if these kinases and transcription factors are connected within the human phosphorylation network and impact biological processes remain open questions that need to be addressed by additional computational approaches. Over the past decade, numerous mechanistic modeling approaches primed by prior knowledge emerged as robust aids in comprehending the complexities of the cell signaling^15^. These approaches use pre-existing information, annotated in public repositories^16–18^, about regulatory interactions among proteins, to establish a ground structure of the signaling network. Subsequently, they incorporate (multi)-omics data to generate a snapshot describing the main molecular mechanisms occurring in a specific condition (*mechanistic model*). Mechanistic models have been shown to be highly effective for studying cancer progression or drug response and for discovering novel biomarkers^19–22^. For instance, the COSMOS pipeline has been used to generate mechanistic hypotheses from multi-omics data, including metabolomics, in patients with clear cell renal cell carcinoma (ccRCC)^23^. In general, mechanistic models aim to bridge the gap between the vast omics datasets and the phenotypic outcomes observed in biological systems. However, models usually contain many nodes and edges, and this complexity hampers their functional interpretation. To tackle this issue, most of the methods use the manual exploration of the model guided by the functional enrichment analysis^19,23^; as an alternative, other tools, such as HiPathia^21^, decompose pathways into functional circuits ending on phenotypes. Finally, we recently developed ProxPath^24^, a graph-based tool designed to estimate the regulatory impact of proteins on phenotypes annotated in SIGNOR^17^.

What is still missing is a strategy that integrates all these procedures (protein activity estimation, network reconstruction, and phenotypic interpretation) in a unified pipeline capable of drawing from multi-omics data a coherent picture depicting the signaling events that eventually impact hallmark phenotypes.

**Fig. 1:**
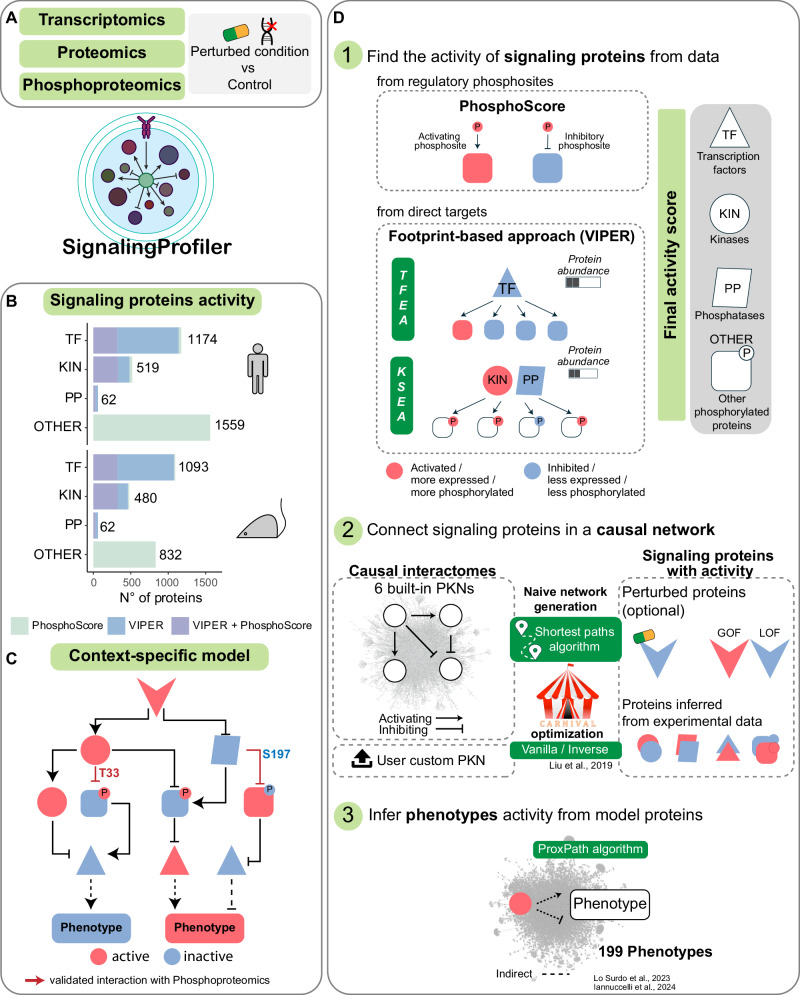
*SignalingProfiler* 2.0 pipeline. **A** *SignalingProfiler* 2.0 input consists of multi-omic data collected from perturbed and control conditions (e.g., disease/ treated vs control). **B** Coverage of *SignalingProfiler* 2.0 inferable signaling proteins in human and mouse datasets, categorized by molecular function (TF transcription factors, KIN kinases, PP phosphatases, and OTHER other molecular functions). **C** *SignalingProfiler* 2.0 final output illustrates the remodeling of the signal, linking user-defined perturbed nodes (optional) with inferred proteins, and ultimately leading to relevant phenotypes. Node activities are coherent with the sign of the edges (red and blue are active and inactive proteins, respectively). Phosphoproteomics is mapped onto edges (validated interactions with phosphoproteomics). **D** *SignalingProfiler* 2.0 is a three-step modular pipeline. Step 1 derives the activity of signaling proteins from regulatory phosphosites (*PhosphoScore* method) and direct transcripts/phosphopeptides using the VIPER algorithm (*footprint-based* methods)^25^ Step 2 A user-defined set of perturbed molecules/receptors (e.g., targets of a treatment or mutated genes in a disease) is connected to the inferred proteins using a prior knowledge network (PKN) exploiting: (i) a shortest-path algorithm to reduce the dimension of the PKN to the neighborhood of the inferred proteins (naïve network); (ii) the CARNIVAL optimization strategy^19^ that retains only the sign-coherent interactions between proteins (context-specific network). Users can provide custom PKNs. Step 3 The context-specific network is connected to cellular phenotypes using the ProxPath algorithm^24^ and the phenotype activity is obtained by integrating upstream protein activities.

To fill this gap, here we present a newly implemented version (2.0) of *SignalingProfiler*. This is a generally applicable strategy that captures from multi-omics data the signal remodeling in response to perturbations (e.g., diseases, drug treatments, etc.). Specifically, *SignalingProfiler* 2.0 integrates transcriptomics, proteomics, and phosphoproteomics data with the existing knowledge of molecular interactions sourced from databases such as SIGNOR^17^ and PhosphoSitePlus^16^. The final output of our pipeline is a model connecting perturbed proteins (e.g., receptors) to effector proteins, ultimately regulating phenotypes relevant to the user’s biological context (Fig. 1).

The first prototype of *SignalingProfiler* allowed us to uncover mechanisms of drug resistance in drug-resistant leukemia cells^20,22^. The current version of *SignalingProfiler (2.0)* extends its utility to broader contexts, includes novel parameters, advanced functionalities, and incorporates expanded databases, making it a valuable resource for a more agile omics data interpretation and hypothesis generation (Fig. 1). Here we carry out a systematic benchmarking of *SignalingProfiler* 2.0, emphasizing its advanced capabilities and broader applicability in the systems biology and network modeling fields.

## Results

### Pipeline overview

*SignalingProfiler* 2.0 is an R workflow designed to unbiasedly integrate literature-derived causal networks with multi-omics data to deliver context-specific signed and oriented graphs connecting molecular entities (e.g. proteins, complexes, metabolites) and ending up on functional traits (phenotypes) (Fig. 1A–C).

The entire pipeline is freely accessible and available for reuse and interoperability at https://github.com/SaccoPerfettoLab/SignalingProfiler/.

Here we provide a step-by-step description of the method and technical parameters explanation for each step is available in Table 1.

**Table 1:** *SignalingProfiler* 2.0 parameters

**Step 1 Protein activity inference parameters**	
Regulons’ sources	Regulons’ databases for Transcription Factors Enrichment Analysis (TFEA) or Kinase Substrates Enrichment Analysis (KSEA) (Supplementary Fig. 1)
Hypergeometric test*	Boolean, using hypergeometric test on VIPER output to weight the inferred activity according to the number of significantly modulated analytes in the regulon
VIPER correction with proteomics*	Boolean, adjust VIPER output based on proteomics fold-change of analytes. If VIPER returns non-significant modulation but the same modulation is significant in proteomics, include the analyte in VIPER result
Normalize phosphoproteomics*	Boolean, correct phosphoproteomics using proteomics data, reducing the importance of phosphosites equally modulated in both datasets
Phosphosites regulating activity	Boolean, in PhosphoScore computation use regulatory phosphosites that affect only protein activity or both activity and abundance (Supplementary Fig. 1)
**Step 2 Network construction parameters**	
Kinome Atlas integration*	Boolean, indicating if PKN contains kinase-substrate relations from the Ser/Thr Kinome Atlas^28^ (Supplementary Fig. 3)
Include only direct iteractions	Boolean, keeping only direct interactions in the PKN (Supplementary Fig. 3)
Preprocess PKN*	Boolean, excluding interactions between proteins not quantified in experimental data
Naïve network types*	Distinguished by layer numbers (one, two, three, Supplementary Fig. 3)
Shortest path maximum length*	Maximum distance between two set of molecules forming a layer in the naive network (Supplementary Fig. 3)
Include interactions between shortest paths (connect_all)*	Boolean, incorporate interactions among proteins identified along distinct shortest paths
CARNIVAL types*	CARNIVAL algorithms types (inverse, vanilla one-shot, vanilla two-shots, vanilla three-shots) (Supplementary Fig. 3)
**Step 3 Phenotypes inference parameters**	
ProxPath preprocessing*	Boolean, exclude paths between model proteins and phenotypes that contain undetected proteins in experimental data
Protein-phenotype path length*	Path length between model proteins and phenotypes
Z-score statistic*	Statistics (mean or median) utilized for randomization in ProxPath (refer to^25^)
Remove cascades*	Boolean, consider only model proteins independently regulating the phenotype
Weight protein contribution*	Boolean, weight protein activity contribution to phenotype based on the number of regulatory paths
Use CARNIVAL activity*	Boolean, consider only experimentally inferred proteins (derived in Step1) or all network proteins (with CARNIVAL activity) in phenotypic activity computation

### Step 1. Find the activity of key signaling proteins

In this step, *SignalingProfiler* 2.0 derives the activity of key signaling proteins by systematically analyzing transcriptomic and (phospho)proteomic data derived from human and mouse samples. Protein activity estimation includes two main methods:

#### Footprint-based approach

Here, *SignalingProfiler* 2.0 determines the activity of transcription factors, kinases, and phosphatases based on the abundance of their targets (transcripts or phosphopeptides) by integrating our newly developed algorithms and statistical tests (Table 1, asterisks indicate novel implementations) with the VIPER inference method^25^. This process is often referred to as Transcription Factor or Kinase Substrate Enrichment Analysis (TFEA and KSEA, respectively) (Fig. 1D, Step 1).

The relationship between a TF/kinase/phosphatase and its specific set of transcripts/phosphopeptides is referred to as ‘regulon’ and is extracted from public repositories^17^^,18,26–29^. A major implementation in *SignalingProfiler* 2.0 is the import of novel regulons, such as the CollecTRI resource^28^ and the Serine/Threonine and Tyrosine Kinome Atlas^27,29^ (Supplementary Fig. 1A, B).

#### PhosphoScore

This method exploits the modulation of phosphosites in phosphoproteomics data with their impact on protein activity or stability as annotated in PhosphoSitePlus and SIGNOR (Supplementary Fig. 1C, D). Importantly, the PhosphoScore methodology allows us to extend our analysis to distinct types of molecular entities: 30% of the proteins with a regulatory phosphosite available in *SignalingProfiler* 2.0 are TF/kinase/phosphatase, the remaining 70% exhibit different GO molecular functions, including, but not limited to, ubiquitin-ligase, GTP-ase, and membrane transporter activities (Supplementary Fig. 1E).

Thanks to the integration of multiple resources and the combination of PhosphoScore and footprint-based methods, the coverage of *SignalingProfiler* 2.0 is greatly expanded: a user can potentially infer nearly the entire kinome (519 and 480 kinases for human and mouse), 62 phosphatases, and over one thousand transcription factors and other signaling proteins (Fig. 1B). Remarkably, the modular nature of the pipeline allows users to feed *SignalingProfiler* 2.0 with the three datasets simultaneously (transcriptomics, proteomics, and phosphoproteomics) or with only a selection of them.

### Step 2. Connect signaling proteins in a causal network

The next step of the pipeline is the reconstruction of the molecular interactions between the modulated signaling proteins, by accessing literature-derived causal networks (Fig. 1D, Step 2). This step includes (i) the search for connections between modulated molecules detected in Step 1 within a compendium of available interactions in a prior knowledge network (PKN) and (ii) the optimization of the final model.

#### The PKNs

*SignalingProfiler* 2.0 offers six categories of prior knowledge networks (PKNs), organized by organism (human or mouse) and covering signaling pathways and post-translational modifications (direct interactions) as well as gene regulation (mostly indirect interactions) derived from public resources^16,17^ (Supplementary Fig. 2 and Supplementary Fig. 3A). Every PKN is a graph built of causal interactions represented according to the activity-flow model. Briefly, every interaction is binary, directed (has a regulator and a target of the regulation), and signed (representing either an up- or a down-regulation). The PKNs contain up to 60,807 connections (Supplementary Fig. 3A) linking a wide range of molecular entities, including proteins, fusion proteins, metabolites, and complexes (Supplementary Fig. 2). Overall, the assembly of these six categories provides a balanced and comprehensive approach to Prior Knowledge Networks, offering a mix of manually curated data, cross-species comparisons, flexibility in computational demands, and the option for customization based on specific research goals. Furthermore, *SignalingProfiler* 2.0 offers ready-to-use strategies to query public resources (SIGNOR, PhosphoSitePlus, OmniPath) and to assemble custom PKNs in a *SignalingProfiler* 2.0 compliant format.

#### The naïve network

*SignalingProfiler* 2.0 allows to progressively make the PKNs context-specific, retaining only interactions in the current knowledge that are responsible for the modulation of TFs, kinases, phosphatases, and other signaling proteins (Step 1). Users have the possibility to embed in the network a set of starting perturbed nodes, which can be proteins whose activity is impacted upon genetic or pharmacological perturbation (e.g., a drug-target, a mutated protein, or ligand-stimulated receptor) (Fig. 1D, Step 2).

First, we allow the user the possibility to remove the interactions that do not involve genes or proteins expressed in the samples (*PKN preprocessing)*. Subsequently, we provide a modular framework to identify the regulatory paths linking the perturbed nodes to transcription factors, resulting in distinct layouts, defined as one-, two-, or three-layered networks (Supplementary Fig. 3B). These layouts arise from the integration of three hierarchical layers: in the first, we retrieve connections bridging perturbed node(s) to kinases and phosphatases (1st layer); in the second we connect kinases/phosphatases to other, undefined signaling proteins (2nd layer); finally, in the third, we link the latter to transcription factors (3rd layer) (Supplementary Fig. 3B).

#### The optimization

The naïve network undergoes optimization upon protein activity through the application of the Integer Linear Programming (ILP). Within the *SignalingProfiler* 2.0 framework, we have incorporated two flavors of the CARNIVAL algorithm, namely Vanilla or Standard CARNIVAL (*StdCARNIVAL*) and Inverse CARNIVAL (*InvCARNIVAL*)^19^. The CARNIVAL algorithm is developed to identify the smallest sign-coherent subnetwork, connecting as many deregulated proteins as possible. To enhance the comprehensiveness of the generated model, we have implemented a novel optimization feature that entails the execution of multiple m (multi-shot) for each layer of the model, producing subparts of the final model. Subsequently, these subparts are combined to form a more expansive and richer representation (Supplementary Fig. 3C–F).

The result of these steps is a mechanistic model that can be explored at the phosphorylation-resolution level (Fig. 1C).

### Step 3. Hallmark phenotypes inference for functional interpretation

An important novelty of *SignalingProfiler* 2.0 is the implementation of the PhenoScore algorithm that infers from the model the regulation of hallmark phenotypes (Fig. 1D, Step 3). Specifically, it incorporates and adapts our in-house ProxPath method^24^, a graph-based algorithm designed to measure the functional proximity of a list of gene products to target pathways and phenotypes, using causal interactions annotated in SIGNOR. The PhenoScore algorithm averages the activity of phenotype upstream regulators in the model and uses this value as a proxy of the activation level of phenotypes (Table 1).

In summary, *SignalingProfiler* 2.0 offers information on ~200 distinct phenotypes (e.g., Proliferation, Apoptosis, G2/M phase transition, etc.) that can be incorporated into the model (Fig. 1C).

Given the importance of prior knowledge in the performance of *SignalingProfiler* 2.0, we set out to regularly update information from source databases, or, in alternative, allow users to use custom datasets.

### Benchmarking strategy

*SignalingProfiler* 2.0 is a versatile tool composed of three steps that can be used independently or in combination. Each step provides a variety of functions with customizable parameters, allowing the user to choose prior information, statistical methods, network generation approaches, and techniques for multi-omic data integration (Table 1).

**Fig. 2:**
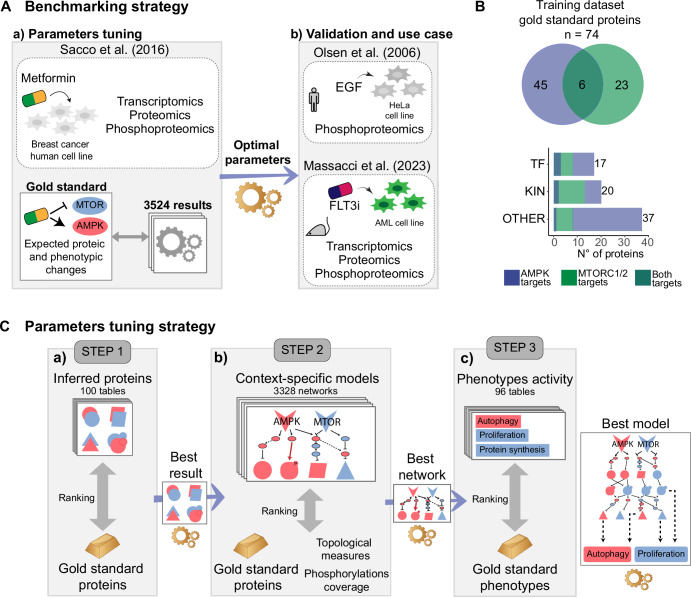
Benchmarking strategy. **A** *SignalingProfiler* 2.0 parameters tuning strategy exploiting multi-omic data of breast cancer cells treated with metformin^30^ (**A**) and best parameters validation on human^39^ and murine multi-omic datasets^20^ (**B**). **B** Manually-curated list of 74 proteins of AMPK (blue), mTOR (green) pathways, or both (dark blue) with their expected activity after metformin treatment (*training dataset protein gold standard*). **C** *SignalingProfiler* 2.0 parameters tuning strategy by testing any possible technical parameters and choosing the best result as input for the following step.

To objectively determine default parameters for future users and systematically assess the performance of *SignalingProfiler* 2.0, we implemented a benchmarking strategy consisting of two parts: (1) *parameters tuning*, where we identify the top-performing technical setting for each step of the pipeline, and (2) *parameters validation* on two independent datasets (Fig. 2).

#### Parameters tuning and performance evaluation

The *parameters tuning* was a standardized evaluation process in which we tested any possible combination of functional parameters of *SignalingProfiler* 2.0 (3524 conditions) (Supplementary Data 2), thus identifying the best parameters to set as defaults (Fig. 2C). Briefly, we took advantage of our previously published transcriptome, proteome, and phosphoproteome dataset of breast cancer cells upon treatment with metformin^30^, whose molecular targets (the mammalian target of rapamycin, mTOR, and the AMP-activated protein kinase, AMPK) and phenotypic impact are well characterized^31–37^ (Fig. 2C). To systematically evaluate the performance of each parameter in recapitulating the metformin-induced signaling rewiring, we manually compiled a literature-derived gold standard. This is a list of known downstream effectors and phenotypes impacted by metformin with their expected activity. The so-generated *protein* and *phenotypic gold standard* accounted for 74 proteins, including 17 transcription factors, 20 kinases, and 10 phenotypes (Fig. 2B, Supplementary Data 1).

#### Performance of parameters in protein activity inference (Step 1)

**Fig. 3:**
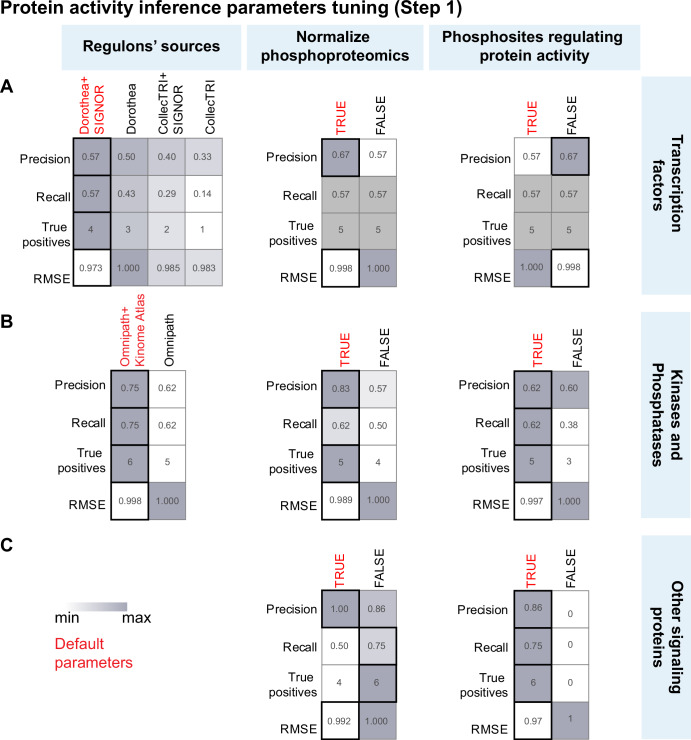
*SignalingProfiler* 2.0 protein activity inference parameters tuning (Step 1). **A**–**C** For each parameter, the average Precision, Recall, Number of true positives, and Root Mean Squared Error (RMSE) with respect to the protein gold standard are reported across 64, 32, and 4 conditions for transcription factors (**A**), kinases/phosphatases (**B**), and other signaling proteins (**C**), respectively. Optimal parameter values are reported in red. White and dark gray represent minimum and maximum parameter values.

Here, we tested 100 distinct combinations of different reference databases (Supplementary Fig. 1A) and technical parameters (Supplementary Data 1 and 2) in inferring protein activity from the training dataset (Supplementary Data 3). Subsequently, by comparing these results with the *protein gold standard*, we systematically assessed the precision, recall, and Root Mean Squared Error (*RMSE*) associated with each combination (Fig. 3), to ultimately identify the optimal set of parameters resulting in an accurate and complete list of modulated proteins (Fig. 2C, panel a, and Supplementary Data 4).

##### Transcription factors

The procedure enabled us to infer the activity of up to 7 out of 17 (40%) transcription factors in the *protein gold standard* (Supplementary Fig. 4A). Our systematic comparison of parameter combinations showed that the choice of *Regulons’ sources* was the most influential parameter, as it positively impacts all the selected performance metrics (Fig. 3A and Supplementary Fig. 4D). In contrast, other parameters, like the *Hypergeometric test* and *Normalize Phosphoproteomics*, displayed a milder impact. Still, these parameters tended to strengthen the signal by reducing the RMSE (Supplementary Fig. 4D).

##### Kinases

The process of inferring kinases resulted in the identification of up to 8 out of 20 (40%) kinases from the gold standard (Supplementary Fig. 4B). In general, the *Regulons’ source* choice had a strong impact on the performance, where integrating Omnipath and Ser/Thr Kinome Atlas increased both Precision and Recall. In addition, other parameters, such as *Normalize Phosphoproteomics*, the *Hypergeometric test*, and *VIPER correction with Proteomics* improved all the metrics, emphasizing the importance of incorporating these novel functionalities in *SignalingProfiler* 2.0 (Fig. 3B and Supplementary Fig. 4D).

##### Other signaling proteins

Among 30 of the non-TFs/kinases in the *gold standard*, eight were identified (27%) (Supplementary Fig. 4C). This benchmarking underscored the importance of the *Normalize Phosphoproteomics* parameter and utilization of phosphosites that regulate activity rather than quantity (*Phosphosites regulating protein activity* parameter) to guarantee minimal RMSE and enhanced precision when using the PhosphoScore algorithm (Fig. 3C).

Overall, the best combination of parameters led to the inference of 23 transcription factors, 41 kinases, 3 phosphatases, and 25 other signaling proteins (Supplementary Fig. 5A and Supplementary Data 5) and was used as an input for Step 2 (Fig. 2C, panel b). As expected, integrating the PhosphoScore method with footprint-based analyses expanded the number of inferred proteins (Supplementary Fig. 5B) while maintaining a high level of agreement with the gold standard (Supplementary Fig. 5C). Remarkably, our pipeline enabled us to catch among the most highly modulated proteins many members of the gold standard (Supplementary Fig. 5A, starred proteins).

To evaluate the robustness of the analysis, the best combination of parameters was also tested over a set of partially degraded experimental data, and regulons, generated by randomly shuffling for 100 times an increasing number (25, 50, 100%) of entries. We next compared each *SignalingProfiler* 2.0 result against the protein gold standard. In both cases, there was a significant decline in precision, recall, and true positives, while false negatives increased. Interestingly, the predictions were extremely sensitive to the regulons’ randomization, where 50 and 100% of the regulons’ shuffling produced no results (Supplementary Fig. 6A, B).

#### Performance of parameters in Network construction (Step 2)

**Fig. 4:**
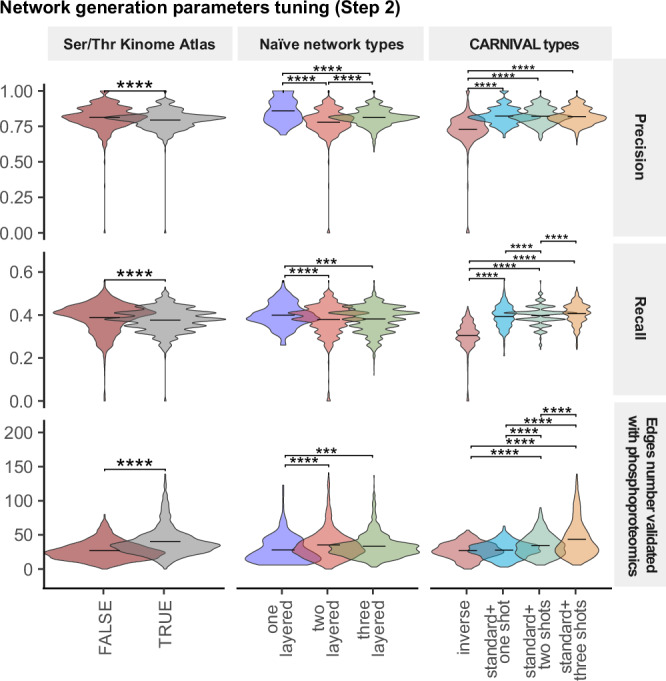
SignalingProfiler 2.0 network generation parameters tuning (Step 2). Violin plots reporting the impact of Ser/Thr Kinome Atlas integration in the prior knowledge network, number of layers in the naïve network, and CARNIVAL flavors and types on Precision and Recall with respect to the gold standard and on the number of interactions validated with phosphoproteomics data, across 2989 generated models. The black line indicates the average *y* value. Statistical significance was computed with *T*-test statistical analysis (*p-value < 0.05, **<0.01, ***<0.001, ****<0.0001).

A key challenge in multi-omics data integration is extracting the cause-effect relationships underlying the experimental data. Translated to our training dataset, this task attempts to address the specific molecular events triggered by metformin treatment. To this aim, we extracted the direct and indirect connections linking the mTOR protein and AMPK complex to the proteins modulated in their activities through any possible framework in Step 2 of *SignalingProfiler 2.0* (Supplementary Fig. 3). This process involved the screening and the evaluation of 3328 possible resulting networks (Table 1 and Supplementary Data 2), by ranking them according to a combined score, that considers elements such as the consistency with the protein gold standard and topological graph metrics (see Methods) (Fig. 4, Supplementary Figs. 7, and 8).

Overall, the networks were obtained from 2989 runs over 3328, with an average computation time of 200 s (Supplementary Fig. 8A and Supplementary Data 6). As shown, the integration of the Ser/Thr Kinome Atlas into the prior knowledge network, as well as the usage of two- and three-layer naïve network types, led to an increased computation time and dimensionality and, as expected, increased coverage of metformin-dependent phosphorylation events (Fig. 4, Supplementary Fig. 7 and Supplementary Data 6).

We also benchmarked the two types of CARNIVAL differentiated by the usage of starting perturbed nodes as constraints. The *invCARNIVAL* required increased computational time (Supplementary Figs. 7 and 8A) and returned smaller networks with reduced precision and recall (Fig. 4 and Supplementary Fig. 7). Moreover, due to the limited constraints and the complexity of the basic network, only 3% of the models generated by *inv*CARNIVAL correctly inferred both mTOR and AMPK, whereas 55% of them inferred only one of them.

On the other hand, the *stdCARNIVAL* returned larger networks with the three-shot optimization outperforming the one- and two-shot ones, in the number of nodes and phosphorylation events, with little impact on the computation time (Fig. 4, Supplementary Fig. 7 and Supplementary Data 6).

Overall, the quality of the models with respect to the gold standard was satisfactory, with an average precision and recall of 0.75 and 0.35, respectively (Fig. 4 and Supplementary Fig. 8).

We ranked the models according to the combined score (Supplementary Fig. 8G and Supplementary Data 6) and set as default the parameters that mostly contributed to the combined score (Supplementary Data 6, **in red**). The top-quality network (Network1554) accounted for 99 nodes and 219 edges and recapitulated the expected mTOR pathway inactivation and AMPK pathway activation upon metformin treatment (Supplementary Fig. 9)^30^.

To investigate the robustness of *SignalingProfiler* 2.0 also in network construction, we generated three classes of randomized PKNs, obtained by randomly shuffling an increasing number (25%, 50%, and 100%) of edges using the BiRewire tool^38^, which generates random networks by preserving, for each node, its signed/directional degree. Next, we evaluated the impact of the PKN shuffling on the mechanistic power of the model by analyzing the number of interactions representing phosphorylation events caught by phosphoproteomic data. As depicted in Supplementary Fig. 6C, their numerosity already decreased at 25%, greatly reducing the model’s mechanistic relevance for experimental data interpretation.

#### Performance of parameters in phenotype inference (Step 3)

**Fig. 5:**
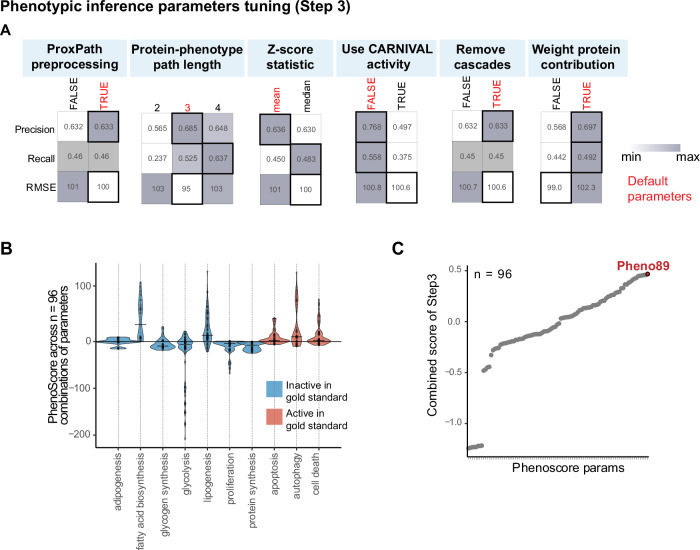
*SignalingProfiler* 2.0 phenotypic inference parameters tuning (Step 3). **A** For each Step 3 parameter, the average Precision, Recall, and Root Mean Squared Error (RMSE) with respect to the *phenotypic gold standard* (Supplementary Data 1) are reported across 96 conditions. Optimal parameter values are reported in red. White and dark gray represent minimum and maximum parameter values. **B** Violin plot illustrating the inferred activity distribution of 10 metformin-treatment-associated phenotypes across the 96 technical conditions of the benchmarking of *SignalingProfiler* 2.0 Step 3. The color of each violin corresponds to the expected activity in the *phenotypic gold standard* (Supplementary Data 1). **C** Scatter plot displaying the distribution of the combined score across each of the 96 technical conditions evaluated to assess their fit to the *phenotypic gold standard* (Supplementary Data 1).

Finally, we used the top-quality network derived from Step 2 as input for the inference of phenotypic outcomes. To note, the PhenoScore algorithm considers various modalities (Table 1), resulting in a total of 96 potential outcomes that were systematically compared to the *phenotypic gold standard* (Fig. 2C, panel c, Supplementary Data 1, and Supplementary Data 7). Briefly, PhenoScore parameters, such as the *ProxPath preprocessing* and *Remove cascades*, had a clear impact on Precision and RMSE, whereas the others showed ambiguous results (Fig. 5). The median phenotypic inferred activity across the 96 conditions aligned with the expected (with few exceptions), suggesting the overall reliability of the algorithm (Fig. 5B). Since the evaluation of individual parameters was not decisive in the default choice, we used an aggregated score to rank the results (see Methods) and the top 10 settings were selected as default (Supplementary Data 7). Then, we used the most accurate prediction of the *phenotypic gold standard* (Fig. 5C) to create a final model (109 nodes and 298 edges) depicting the metformin-induced signaling axes impacting the selected phenotypes (Supplementary Fig. 10, and Supplementary Data 8). Interestingly, in this final model, metformin results in the activation of death-associated pathways (e.g., apoptosis and cell death) and autophagy (the most characterized phenotypic hallmark of mTOR inhibition)^31^, and in the inhibition of proliferation and biosynthetic pathways (e.g., protein synthesis) (Supplementary Fig. 10 and 11).

##### Independent validation

**Fig. 6:**
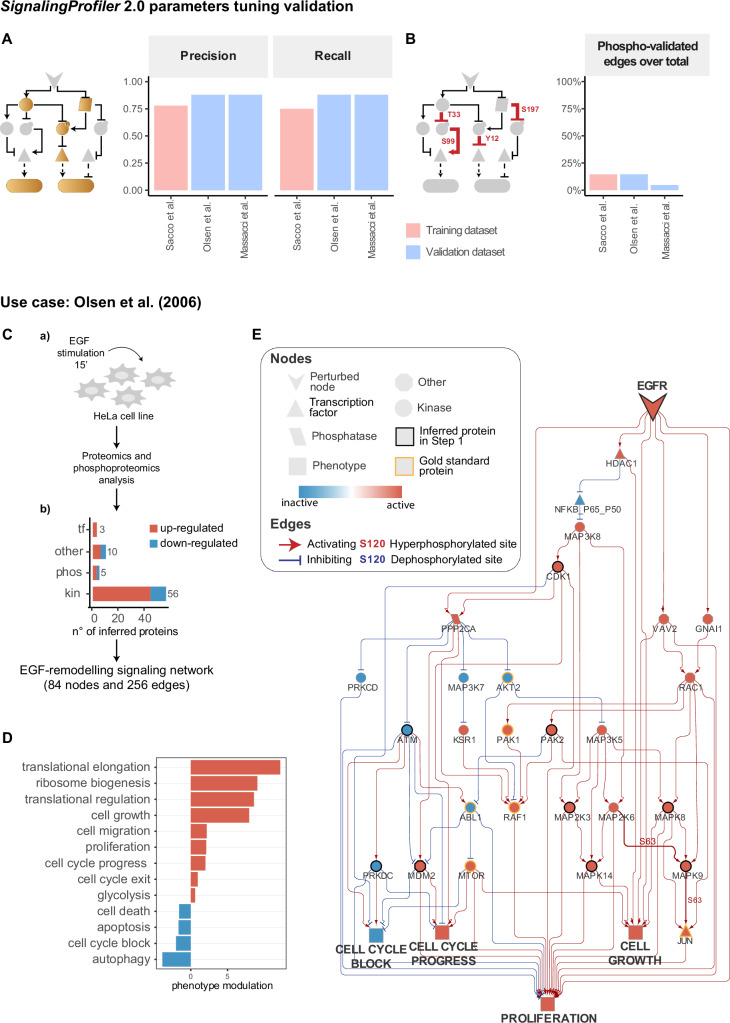
*SignalingProfiler* 2.0 parameters tuning validation and EGF use case. **A**, **B** For training and validation datasets, the bar plot reports the Precision and Recall with respect to the *protein* and *phenotypic gold standard* (**A**), and the percentage of edges representing experimentally quantified phosphorylations (**B**). **C**–**E** *SignalingProfiler* 2.0 application on data from EGF-stimulated HeLa cell lines. **C** Cartoon of experimental strategy (panel **a**) and *SignalingProfiler* 2.0 results (panel **b**). **D** Bar plot of the phenotypic modulation upon EGF stimulation inferred by *SignalingProfiler 2.0*. Blue and red bars represent inactive and active phenotypes, respectively. **E** Functional circuit extracted from the EGF-model impacting cell cycle and proliferation.

With the dual aim of confirming that the optimal parameters selected were not suffering from an ‘overfitting’ effect and could find applicability on a broader set of input data, we cross-validated *SignalingProfiler* 2.0 on two independent and heterogeneous datasets (Fig. 2A, B, Fig. 6A, B).

The first dataset reported the HeLa cell line phosphoproteomic profile modulation upon EGF treatment^39^ (Fig. 6C). The second consisted of multi-omics data from a murine acute myeloid leukemia (AML) cell line treated with ac220, a tyrosine kinase inhibitor (TKIs) targeting the *FMS‐like tyrosine kinase 3* (Flt3), as described in our previous work^20^ (Supplementary Fig. 12).

For both datasets, we manually compiled a list of expected protein and phenotypic activities (gold standard) (Supplementary Data 9). As shown in Fig. 6A, B, *SignalingProfiler* 2.0 (default options) displayed comparable performance in terms of accuracy and phosphorylations coverage for both datasets, confirming the broad applicability of the approach (Supplementary Data 10, 11 and 12).

### Biological Insights and Exemplary Results with SignalingProfiler 2.0

*SignalingProfiler* 2.0 is a tool for extracting molecular hypotheses and functional insights into the molecular mechanisms of signal transduction from multi-omics data. By integrating and analyzing diverse data types, *SignalingProfiler* 2.0 can uncover complex interactions and regulatory networks, providing a comprehensive understanding of cellular signaling pathways. Here we aim to picture a clear example of the results of *SignalingProfiler* 2.0 for potential users. To this scope, in Fig. 6C, we summarized the output from the analysis of Olsen et al.^39^.

Briefly, from phosphoproteomics data, we inferred the activity modulation upon EGF stimulation of 56 kinases, 5 phosphatases, 3 transcription factors, and 10 other signaling proteins (Fig. 6 and Supplementary Data 10). The resulting signaling network encompassed 84 nodes and 256 edges, where 14% were experimentally detected phosphorylation events (Fig. 6B). In agreement with findings from the original publication, *SignalingProfiler* 2.0 successfully identified proteins downstream of EGF, including BRAF, RAF1, MAPK1-3 (ERK1/2), MAPK14 (p38) kinases, along with ATF7 and JUN transcription factors (Supplementary Data 10). The resulting EGF network accurately recapitulated signaling cascades associated with pro-proliferative pathways activation and cell death-related pathways inhibition (Fig. 6D). Notably, the molecular circuits leading to the deregulation of individual phenotypes can be inspected as individual or as grouped maps of regulatory interactions at the phosphorylation-resolution level (Fig. 6E). As an example, in Fig. 6E we highlighted the subnetwork combining the axes impacting Cell Growth, Proliferation, Cell Cycle Block, and Cell Cycle Progression. The circuit not only recapitulated known signaling cascades (e.g. the one involving the MAPK family members) but also shed light on potential crosstalk between different axes, allowing users to formulate novel hypotheses.

Overall, these results highlighted the reliability of the benchmarking strategy and *SignalingProfiler* 2.0 applicability on a broader set of input data. The findings discussed in this manuscript demonstrated that *SignalingProfiler* 2.0 is a powerful tool for extracting molecular hypotheses from multi-omics data and for identifying functional circuits that influence phenotypes.

## Discussion

In this paper, we thoroughly present *SignalingProfiler* 2.0, a method to create mechanistic context-specific networks of signaling remodeling and to identify functional circuits impacting phenotypes.

Here, we show that *SignalingProfiler* 2.0 is a modular pipeline that allows users (i) to unbiasedly derive the activity of proteins from the integration of proteogenomic data with prior knowledge information deposited in public repositories; (ii) to connect the identified proteins to generate a coherent network that explains nodes’ change in activity; (iii) to estimate the activation level of hallmark phenotypes and integrate them in the final model. As compared to *SignalingProfiler* 1.0^20^, the 2.0 version incorporates: (i) extended background databases, such as CollectTRI^28^ and Ser/Thr Kinome Atlas^27,29^, (ii) increased coverage and accuracy of protein activities’ prediction, (iii) PKN browsing methods, optimization strategies, and PhenoScore inference with ProxPath^24^. Indeed, it is possible to estimate the activity of >3300 proteins, including, but not limited to, kinases, phosphatases, and transcription factors, and map up to 200 cellular phenotypes onto the final model (Supplementary Data 13). This represents an effective strategy of feature reduction and a valuable resource for omics data interpretation and hypothesis generation in diverse biological contexts.

To systematically identify the parameter settings that optimize the performance of *SignalingProfiler* 2.0, we employed a benchmarking approach consisting of a *parameter tuning phase* on a training dataset^30^ and a subsequent *validation phase* on murine^20^ and human^39^ datasets. Notably, the parameter settings determined during the training process exhibited comparable performance (Fig. 6A) when applied to the validation datasets, demonstrating their broad applicability and limited risk of ‘overfitting’.

The *validation phase* also highlighted an important feature of *SignalingProfiler* 2.0 which is flexibility. Flexibility on required multi-omics data (e.g. users can employ only transcriptomic or phosphoproteomic data); flexibility on the organism choice (mouse and human data are accepted) and flexibility on the type of perturbed nodes, since we include relations that are both signaling or transcriptional; also, thanks to its modular structure, users can use only a limited number of steps of the pipeline and, possibly, to integrate *SignalingProfiler* 2.0 with other methods for protein activity estimation and network optimization.

Indeed, *SignalingProfiler* 2.0 is not the sole method to generate a mechanistic network from omics data. Here, we report a systematic comparison of *SignalingProfiler* 2.0 with a panel of similar methods, released from 2017 to 2022^19,21,23,40–44^ (Table 2). Our analysis reveals that *SignalingProfiler* 2.0 is: (i) one of the few techniques directly annotating meta-information about the molecular function at node/protein levels, (ii) is the sole tool capable of estimating the activity of proteins, aside from kinases and phosphatases, from the phosphoproteomic data and (iii) is the sole approach together with CausalPath^42^ combining proteomics in the analysis and, apart from HiPathia^21^, integrating phenotypes with their activation status into the ultimate model to unbiasedly derive functional circuits.

**Table 2:** Qualitative comparison of *SignalingProfiler* 2.0 and existing methods

*Omics data properties*		*SignalingProfiler* 2.0	COSMOS	CausalPath	TPS	CARNIVAL	HiPathia	CausalR	NicheNet	KPNN
**Omics layers**	Transcriptomics	**X**	X	X		X	X	X	X	X
	Proteomics	**X**		X						
	Phosphoproteomics	**X**	X	X	X					
	Metabolomics		X							
**Biological resolution**	Bulk/Pseudo-bulk	**X**	X	X	X	X	X	X	X	
	Single-cell						X		X	X
**Omics data used as**	Measurements (observations)				X		X			X
	Statistical scores (contrast/correlation)	**X**	X	X	X	X		X	X	
**Additional input**	Does not use additional inputs		X				X	X		X
	Accepts additional inputs	**X**		X		X			X	
	Requires additional inputs				X					
**PKN properties**										
**Sign**	Directed				X				X	X
	Activations/Inhibitions	**X**	X	X	X	X	X	X		
**Size**	Pathways						X			
	Large networks	**X**	X	X	X	X		X	X	X
**Biological content**	Protein signaling interactions	**X**	X	X	X	X			X	X
	Gene regulatory interactions	**X**	X	X			X		X	X
**Method properties**										
**Omics to PKN**	Direct mapping to nodes			X	X		X	X	X	X
	Indirect mapping to nodes	**X**	X			X				
**Not‐measured nodes**	Estimates unmeasured nodes state	**X**	X		X	X	X	X		X
	Includes unmeasured nodes in output	**X**	X		X	X	X	X	X	X
**Algorithm type**	Edge filtering and shortest path	**X**		X				X	X	
	Recursive signal propagation and heat diffusion						X			
	Integer linear programming	**X**	X		X	X				
	Neural networks									X
**Final network properties**										
	Includes user-defined perturbed nodes	**X**				X	X			
	Includes phenotypes	**X**					X			
	Includes meta-information on nodes	**X**	X			X			X	
	Includes meta-information on edges regarding phosphorylation events	**X**		X	X					

Finally, the comparison with other methods highlights some of the limitations of our pipeline. Compared to tools such as COSMOS^23^, *SignalingProfiler* 2.0 does not include metabolomic data. At the present state, additional types of regulation such as epigenetic, acetylomic, and ubiquitylomic data, which are becoming more popular^45,46^ cannot be integrated into the signaling and represent a future challenge to face. Also, as for all the methods that base their prediction on prior knowledge, *SignalingProfiler* 2.0 suffers from the limited coverage of available information in public repositories: either regulon databases and causal interaction resources are incomplete and offer information for <50% (about 9,000 proteins) of the Uniprot-SwissProt proteome. As an important novel feature to balance this limitation, we also implemented regular updates of the prior knowledge information from source databases to ensure up-to-date data.

In summary, *SignalingProfiler* 2.0 is a versatile and flexible pipeline that efficiently generates mechanistic networks from multi-omics data hierarchically bridging signaling molecules to phenotypic traits. As such, it addresses the emergent need to extract interpretable networks and derive biologically relevant information from complex multi-omics data. We expect that in the multi-omics era, where the proteogenomic characterization of human samples and biopsies are becoming increasingly more available to the public^47,48^, *SignalingProfiler* 2.0 could pave the way to the development of personalized medicine strategies.

## Methods

### PKNs creation

We downloaded all causal interactions available for *Mus musculus* (TaxID = 10090) and *Homo sapiens* (TaxID = 9606) from the SIGNOR and PhosphoSitePlus® resources. SIGNOR 3.0 datasets, retrieved via the REST API, are based on information up to November 2023. Interactions labeled ‘down-regulates,’ ‘up-regulates,’ and ‘form complex’ in SIGNOR were assigned values of −1, 1, and 1, respectively. Interactions involving entities with the TYPE ‘protein family’ in SIGNOR were excluded. Causal phosphorylations from PhosphoSitePlus® were obtained by manually downloading and combining two independent tables: kinase-phosphosite interactions (‘Kinase_Substrate_Dataset.gz’) and the regulatory role of phosphosites on proteins (‘Regulatory_sites.gz’). The tables were joined using the UniProt ID and modified residue as keys. The content of the ‘ON_FUNCTION’ column in PhosphoSitePlus® representing the regulatory role of phosphosites was mapped to values of 1, −1, or 0. These manipulated datasets were merged and filtered to retain interactions with a defined regulatory effect (−1 or 1). For *Homo sapiens*, causal interactions derived from the Ser/Thr Kinome Atlas were added to SIGNOR and PhosphositePlus datasets (see Methods ‘Ser/Thr Kinome Atlas parsing’). UniProt IDs were updated, and primary Gene Names were retrieved using the UniProt database’s REST API. The primary Gene Names of the involved entities were used as keys for each interaction, and multiple UniProt IDs and attributes (e.g., TYPE, DATABASE field of SIGNOR) were collapsed into a single string. We created six Prior Knowledge Networks (PKNs) adding increasingly exclusive filtering criteria: no filtering (PKN2 for human and PKN6 for mouse), removal of indirect interactions representing ‘transcriptional regulations’ (PKN1 for human and PKN5 for mouse), removal of Kinome Atlas interactions (PKN4), and removal of direct interactions not involving proteins (PKN3). The number of nodes and edges of each PKN is shown in Supplementary Figs. 1 and 3. *SignalingProfiler* 2.0 PKNs are available in the R package as built-in objects, but users can also create custom PKNs.

### Ser/Thr Kinome Atlas parsing

We obtained Supplementary Data 4 from the work of ref. ^27^, containing information on 89752 serine (Ser) and threonine (Thr) sites and their probabilities (or percentile) of being phosphorylated by 303 Ser/Thr kinases. We kept phosphosite-kinase relations with a percentile >88 (‘*regulon threshold’*) and 99 (‘*PKN threshold’)*, retaining 3,134,109 and 291,682 relations, respectively.

The ‘*regulon threshold’* of 88 was determined as the median value from the distribution of percentiles of phosphosite-kinase relations documented in SIGNOR or PhosphoSitePlus®. These relations were incorporated into the regulons for kinase inference analysis, with weights assigned proportionally to the percentiles within the range of 0.5 to 0.9.

The ‘*PKN threshold’* of 99 was chosen to keep only the most accurate relationships. We joined this table with the PhosphoSitePlus table on the regulatory role of phosphosites (‘Regulatory_sites.gz’) using the phosphosite as key. As a result, we included 28,012 interactions in the prior knowledge networks, representing relationships between kinases from the Atlas and proteins for which the regulatory phosphosite is known.

Each kinase was annotated with its UniProt ID.

### Benchmarking strategy

We first exploited a *training dataset*^30^ to identify the best-performing technical setting for each step of the pipeline (parameters tuning) and we then validated the optimal parameters on two independent validation datasets, one from human^39^, and one from mouse^20^ (parameters validation).

#### Training and validation datasets preparation

For the training dataset, we downloaded relevant tables from our work as published in ref. ^30^ to build transcriptomic, proteomic, and phosphoproteomic data tables. The so-obtained information was parsed and adapted to make it *SignalingProfiler* 2.0 compliant.

Briefly, the dataset accounted for 9591, 7974, and 15812 quantified transcripts, proteins, and phosphosites. These tables included computed fold-change values among three replicates of both the control and metformin conditions.

For the human validation dataset, we downloaded phosphoproteomic data table from^39^ work. We updated UniProt IDs and phosphopeptide sequences by querying the UniProt database via API. Data was analyzed using the DEP R package (v. 1.16.0). Briefly, we normalized intensities using variance stabilizing transformation (vsn). We kept 22773 phosphosites that had just 1 missing value in at least one condition (thr = 1) and imputed missing data with the ‘MinProb’ (*q* = 0.01) DEP method. The DEP *test_diff* function called *limma* to identify significantly modulated phosphopeptides between EGF stimulation for 15 min and control (*p*-value adjusted <0.05, fold-change threshold = 1).

For the murine validation dataset, we downloaded transcriptomic, proteomic, and phosphoproteomic datasets from our work as published in ref. ^20^.

The three datasets were parsed to make them *SignalingProfiler 2.0* compliant.

#### Normalization of phosphoproteomics over proteomic data

We created a normalized phosphoproteomic dataset (Normalize Phosphoproteomics parameter) by adjusting the fold-change in phosphorylation in response to metformin treatment based on the corresponding fold-change in protein abundance. To achieve this, we calculated the difference between the phosphorylation level of the phosphosite and its associated fold-change in protein abundance. The phosphorylation levels of phosphosites that showed modulation in proteomics with the same direction were reduced, while those with opposite phosphorylation and protein abundance changes were increased. We then computed the Z-score for the new distribution of phosphorylation fold-changes using their mean and we defined corrected fold-changes with an absolute value >1.96 (i.e., *p*-value < 0.05) significant.

#### Protein and phenotypic gold standard creation

We manually curated a list with their expected activity for training and validation datasets.

For the training dataset, a list of 74 proteins with their expected activity modulation to metformin treatment (*protein gold standard*) was compiled from three recent papers^32,33,36,37^ focusing on mTOR and AMPK pathways. Since metformin inhibits mTOR (and activates AMPK), negative and positive targets of mTOR (and AMPK) were set to active and inactive (inactive and active), respectively. Each protein was manually cross-referenced and converted to its primary gene name. The molecular function of each protein was annotated using *SignalingProfiler* 2.0. The resulting gold standard protein list was compared with proteins in *SignalingProfiler* 2.0 databases, including TFEA or KSEA regulons and the PhosphoScore database. Notably, eight proteins were not found in the databases and were consequently labeled as ‘not inferable’ proteins. Additionally, a list of 10 phenotypic traits with their expected modulations upon metformin treatment (*phenotypic gold standard*) was compiled, based on the phenotypic readout from our previous work^30^ and three referenced papers^31,34,35^. The complete training dataset gold standard is available in Supplementary Data 1.

Similarly, for the validation datasets we compiled protein and phenotypic gold standards accounting for 46 and 33 proteins, and 21 and 7 phenotypes with their expected activity in Olsen et al.^39^ and Massacci et al.^20^ datasets, respectively. The complete list and associated references are available in Supplementary Data 9.

#### *SignalingProfiler* 2.0 parameters tuning

We ran the *SignalingProfiler* 2.0 pipeline with all technical parameter combinations. A detailed explanation of *SignalingProfiler* 2.0 functions and parameters is provided in Table 1. Briefly, any test combination of parameters was evaluated by measuring precision, recall, and Root Mean Squared Error (RMSE) using protein and phenotypic gold standard lists.

##### Precision, recall, and RMSE definition

We defined an inferred protein matching and diverging the expected value, as *true* and *false positive*, respectively*. False negatives* were proteins present in the gold standard but not inferred. *True negatives* were proteins with opposite activity than expected and not inferred. We calculated as quality metrics (i) *precision*, the ratio of true positives to the sum of true and false positives, (ii) *recall*, the ratio of true positives to the sum of true positives and false negatives, and (iii) *RMSE*, the squared mean difference between predicted and expected values. The eight ‘not inferable’ gold standard proteins were not considered in the quality metrics computation.

##### Step 1 parameters tuning

*SignalingProfiler* 2.0 independently infers the activity of transcription factors, kinases/phosphatases, and phosphorylated proteins. Transcription factors/kinases/phosphatases can be inferred with footprint-based methods, PhosphoScore, or a combination of both. The parameter combinations for the inference of transcription factors, kinases/phosphatases, and phosphorylated yielded 64, 32, and 4 results, respectively (Supplementary Datas 2 and 3). Each result referred to a unique selection of parameters for the type of regulons/phosphosites database, the usage of the Hypergeometric Test, VIPER correction with proteomics, and correction of phosphoproteomics over proteomics.

The default setting for Step 1 was determined by selecting the result for each molecular function that maximizes precision and recall while minimizing RMSE (Supplementary Data 4 and 5).

##### Step 2 parameters tuning

The network construction step involves 7 main parameters (see Table 1, Supplementary Data 2 for details). All combinations resulted in 3328 possible results, but only 2989 combinations yielded actual networks. Each model was annotated with *computation* time (sum of naïve network computation and CARNIVAL optimization time); *topological* metrics (nodes, edges and components, clustering coefficient, diameter, fit to the power law, maximum path length between end nodes and AMPK, mTOR and Perturbation node created by Inverse CARNIVAL); *biological* metrics, such as the precision, recall and RMSE with respect to the gold standard, and the number of interactions validated by quantified or significant experimental phosphorylations (Supplementary Data 6). We developed a Step 2 combined score defined as follows:

$$\begin{array}{l}{Combined\; scor}{e}_{{step}2}=\left({precision}* 0.5+{recall}* 0.5+{SignRatio}* 0.5+{ClusteringCoefficient}* 0.5\right)\\\qquad\qquad\qquad\qquad\quad-\left({NormTime}* 0.2+{PowerLawFit}\right)\end{array}$$

where *SignRatio* is the ratio between the number of edges that are validated by significant phosphorylation events over the total and the *NormTime* is the ratio between each computation time and its maximum. The best model was selected based on the highest aggregate score (Network1554 with 99 nodes and 219 edges) and we set its parameters as default for Step 2.

##### Step 3 parameters tuning

The phenotypic traits inference considers 6 different parameters (see Table 1, Supplementary Data 2 for details). The ten phenotypes of the phenotypic gold standard were selected, including apoptosis, autophagy, adipogenesis, biosynthesis of fatty acids, glycogen and proteins, proliferation, and glycolysis. We obtained 96 results that were compared to the phenotypic gold standard, and we annotated precision, recall, RMSE, and computation time (Supplementary Data 7). We formulated a Step 3 combined score:

$${Combined\; scor}{e}_{{step}3}=\left({precision}+{recall}\right)-({normRMSE}+{normTime}* 0.5)$$

where *normRMSE* and *normTime* are the ratio of its value and its maximum.

We linked the phenotypes’ values with the highest combined score to their regulators in the Step 2 model, resulting in a final optimized network of 109 nodes and 309 edges (Supplementary Data 8).

The network is publicly available for browsing at: https://www.ndexbio.org/viewer/networks/fa22e724-b54b-11ee-8a13-005056ae23aa. The Step 3 default was set by considering the most represented parameters’ values among the top 10 results.

#### *SignalingProfiler* 2.0 best parameters validation

To validate the parameters tuning result, we run *SignalingProfiler* 2.0 on both validation datasets with the optimal parameters established using the training dataset. For the Olsen et al.^39^ dataset, we inferred 74 proteins (3 transcription factors, 56 kinases, 5 phosphatases, and 10 other signaling proteins) and generated a network of 84 nodes (72 proteins and 12 phenotypes) and 256 edges (Supplementary Data 10). For Massacci et al.^20^ dataset, we predicted the activity of 87 transcription factors, 53 kinases, 8 phosphatases, and 4 other signaling proteins (152 proteins in total) and generated a network of 180 nodes (172 proteins and 8 phenotypes) and 419 edges (Supplementary Data 11). Both networks are publicly available on NDEX (Olsen et al.: https://www.ndexbio.org/viewer/networks/59ab8c7b-0611-11ef-9621-005056ae23aa; Massacci et al: https://www.ndexbio.org/viewer/networks/bde743d2-0613-11ef-9621-005056ae23aa).

For both datasets Precision and Recall with respect to their protein and phenotypic gold standard and the number of experimental phosphorylation interactions in the model was determined and compared to the training dataset results (Supplementary Data 12).

#### Randomization analysis

We generated a set of randomized versions of experimental data, regulons, and PKN where an increasing number of analytes or edges were shuffled (25, 50, 100% of all analytes/edges). To shuffle both regulons and PKN, we exploited *birewire.sampler.dsg* function of the BiRewire (v. 3.26.5) R package, to generate 100 randomized graphs for each percentage independently. We evaluate separately the impact of each randomization. Then, *SignalingProfiler* 2.0 was run for each randomized experimental dataset, regulon, or PKN with the same parameters as the original run. To evaluate the noise sensitivity for the protein inference step of *SignalingProfiler* 2.0 (Step 1) we compared the results with the protein gold standard. For Steps 2–3, we analyzed the coverage in the resulting network of experimentally caught phosphorylations.

#### *SignalingProfiler* 2.0 output visualization

The optimized networks generated by *SignalingProfiler* 2.0 were displayed on Cytoscape using the RCy3 package (v. 2.14.2). Two XML files provided within the *SignalingProfiler* 2.0 R package were used to set the network style in Cytoscape.

The *‘SignalingProfiler* 2.0 *layout’* provides users with a clear and intuitive visual representation of the entire model (used in Fig. 6 and Supplementary Figs. 10–12). On the other hand, the *‘Phosphorylation layout’* (used in Supplementary Fig. 9) allows users to focus specifically on proteins involved in experimentally confirmed phosphorylation events.

### Supplementary information

Supplementary Information

Supplementary Data 1

Supplementary Data 2

Supplementary Data 3

Supplementary Data 4

Supplementary Data 5

Supplementary Data 6

Supplementary Data 7

Supplementary Data 8

Supplementary Data 9

Supplementary Data 10

Supplementary Data 11

Supplementary Data 12

Supplementary Data 13

## Data Availability

No new experimental data was generated as part of this study. The multi-omic data of the three benchmarking datasets are taken from refs. ^20,30,39^. The data in *SignalingProfiler* 2.0 compliant format and the resulting networks are available at https://github.com/SaccoPerfettoLab/SignalingProfiler_Benchmarking.

## Code availability

All code used for *SignalingProfiler* 2.0 benchmarking is available at https://github.com/SaccoPerfettoLab/SignalingProfiler_Benchmarking. *SignalingProfiler* 2.0 R package code is available at https://github.com/SaccoPerfettoLab/SignalingProfiler.
