# Supplementary material for: SignalingProfiler 2.0 a network-based approach to bridge multi-omics data to phenotypic hallmarks
Source: NPJ Syst Biol Appl. 2024 Aug 23;10:95. doi: 10.1038/s41540-024-00417-6 (PMC11343843; doi:10.1038/s41540-024-00417-6)
Supplement: Supplementary file 1 — Supplementary Information [file 41540_2024_417_MOESM1_ESM.pdf]

Supplementary material for Venafra et al.

Supplementary Figures

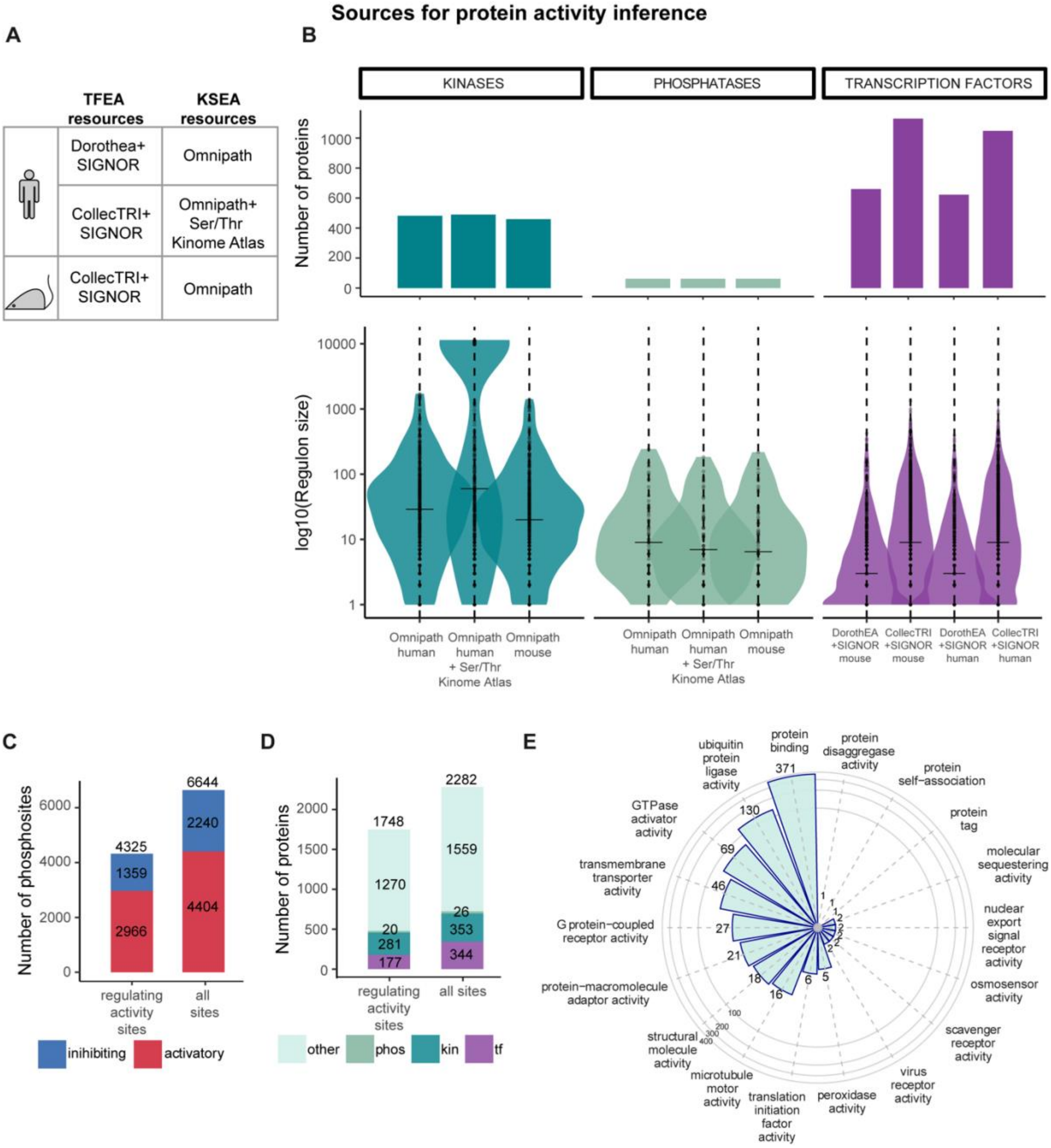

Supplementary Figure 1. ***SignalingProfiler*** **2.0 sources for protein activity inference.**

A-B. *SignalingProfiler* 2.0 regulon sources for footprint-based activity inference of transcription factors (transcription factor enrichment analysis or TFEA) and of kinases and phosphatases (kinase substrates enrichment analysis or KSEA) (**A**) and their associated number of inferable proteins (upper panel) and distribution of the regulon size (lower panel) (**B**). The black line indicates the median regulons' size.

C-D. Number of *SignalingProfiler* 2.0 phosphosites regulating protein activity or stability (**C**) and their associated proteins (**D**), as obtained from SIGNOR (Lo Surdo *et al*, 2023) and PhosphoSitePlus (Hornbeck *et al*, 2012), for the PhosphoScore analysis.

E. GO molecular functions, obtained with gProfiler (Goel *et al*, 2012), associated with proteins having regulatory phosphosites in the PhosphoScore database and that are not kinases, phosphatases, or transcription factors and defined in *SignalingProfiler* 2.0 as "other signaling proteins" (or OTHER).

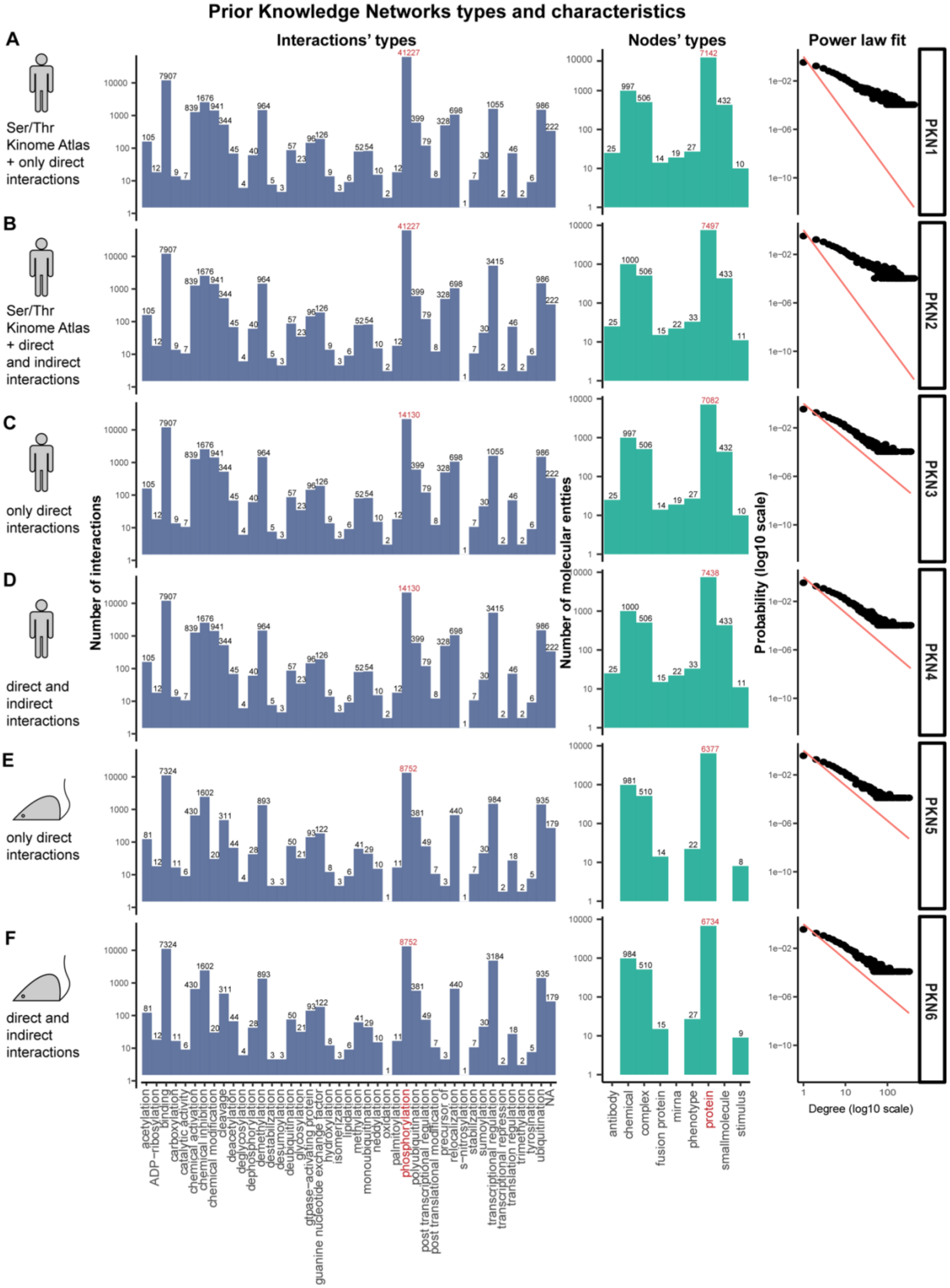

Supplementary Figure 2. The six Prior Knowledge Networks (PKNs) of *SignalingProfiler* 2.0.

A-F. For each PKN: description reporting organism, integrated resource, and type of interactions (*left panel*); bar plot representing the number of interactions for each type of

molecular event (*mid-left panel*); bar plot representing the number of molecular entities for each type (*mid-right panel*); scatterplot of the distribution of the PKN nodes degree in log10 scale and its fit to the power law (red line) (*right panel*).

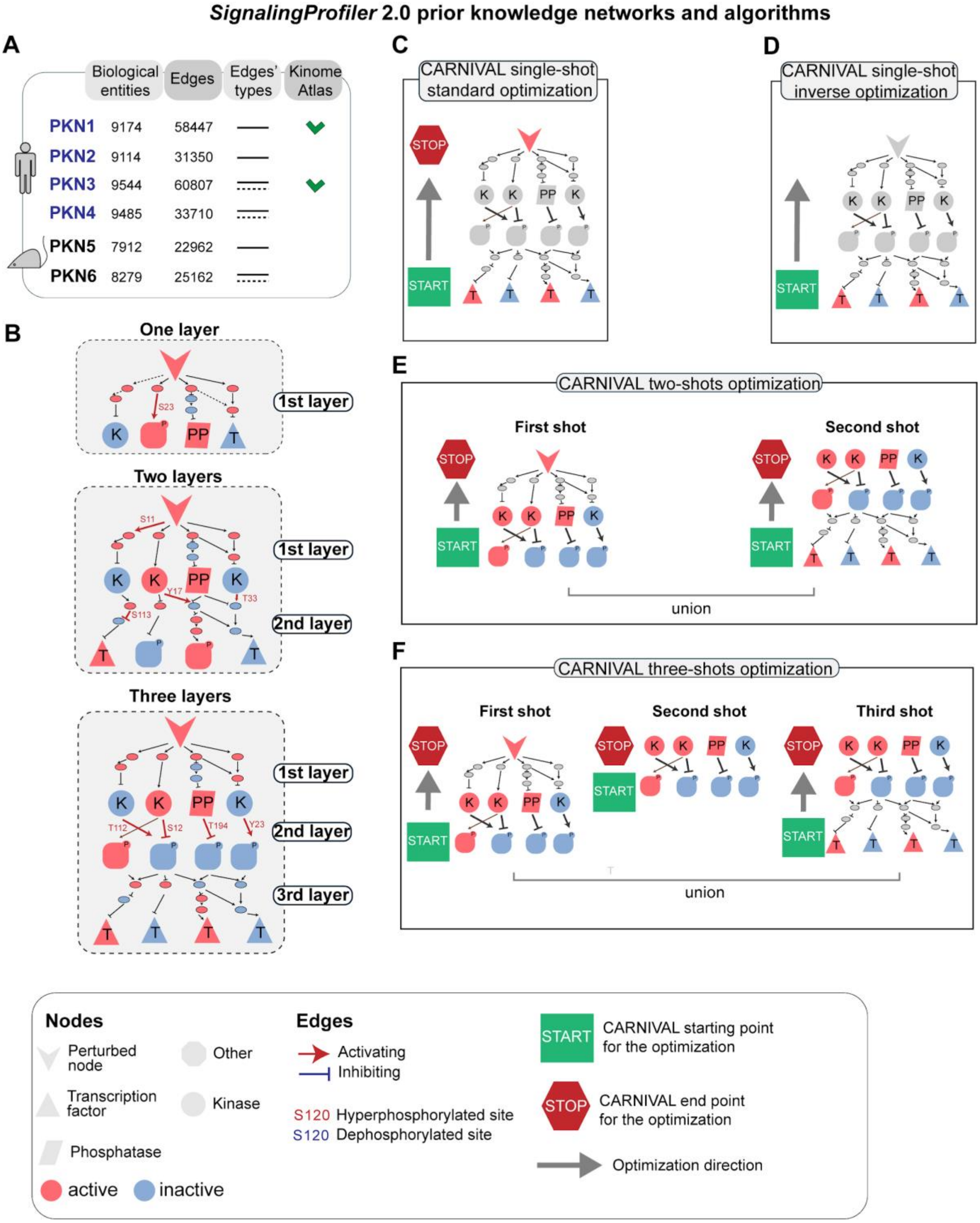

Supplementary Figure 3. ***SignalingProfiler*** **2.0 prior knowledge networks (PKNs) and algorithms for network construction.**

A. Table representing the description and number of nodes and edges of the six prior knowledge networks (PKN).

B. Types of naïve networks that can be created by the shortest path algorithm. In the one-layer network, the perturbed node (starting point) is connected to all inferred proteins without differentiating their molecular functions. The two-layered network connects the perturbed node to kinases/phosphatases/others and then creates a second layer connecting them to transcription factors. The three-layered network adds another layer between kinases/phosphatases and other signaling proteins.

C-F. Different CARNIVAL implementations available in *SignalingProfiler* 2.0. The CARNIVAL algorithm, running in a single iteration, can be executed with (**C**) or without (**D**) a predefined endpoint (or perturbed nodes), denoted as the *standard* and *inverse* CARNIVAL, respectively. Additionally, two multi-shot versions are available in *SignalingProfiler* 2.0. In the two-step optimization, we first optimize from perturbed proteins to kinases, phosphatases, and other signaling proteins. This is followed by the optimization from the latter group to transcription factors, with subsequent union of the optimized sets (**E**). In the three-step optimization, the process involves consecutive runs: from perturbed proteins to kinases and phosphatases, then from kinases and phosphatases to other signaling proteins, and finally from the last set to transcription factors. Then, all the optimized submodels are combined (**F**).

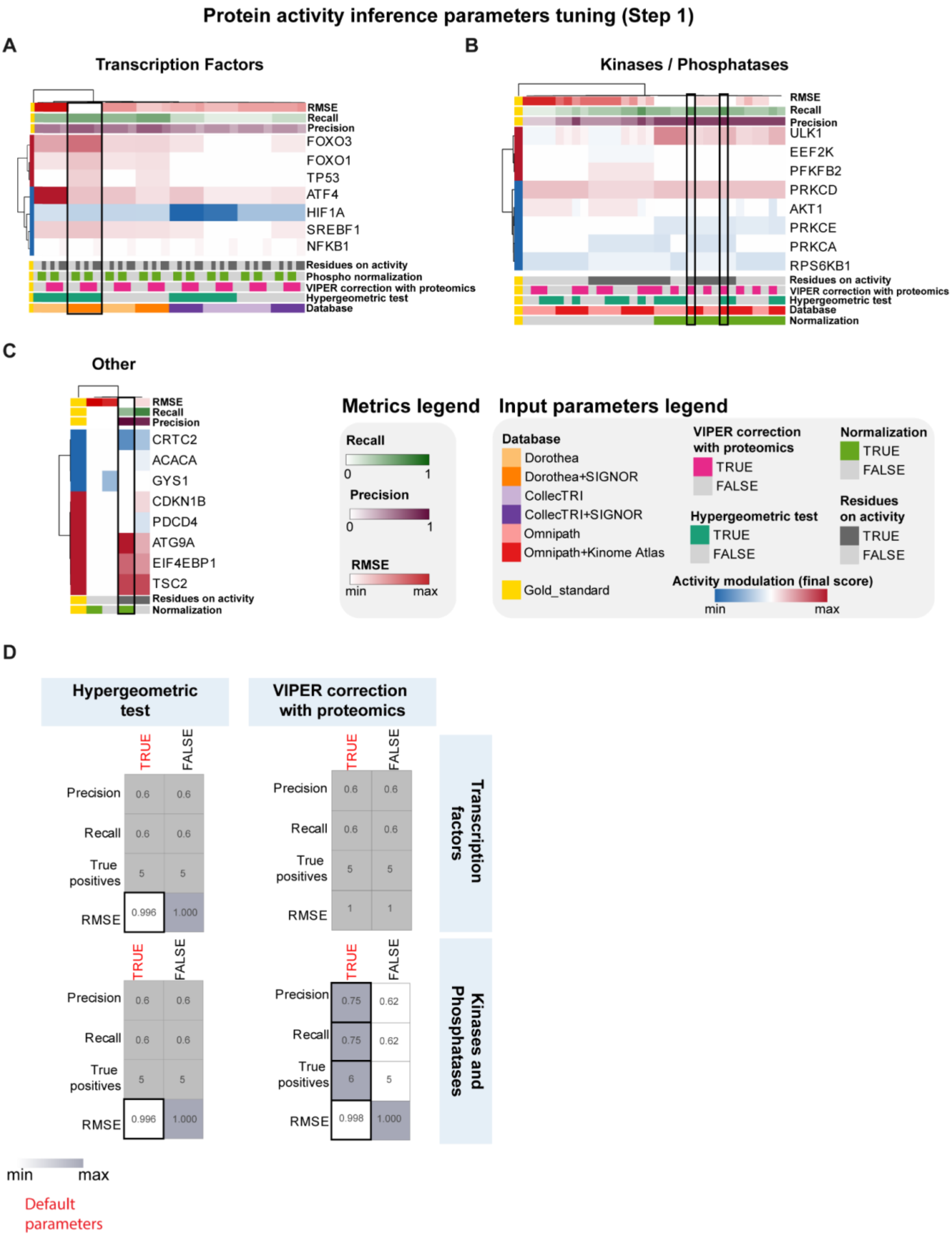

Supplementary Figure 4. ***SignalingProfiler*** **2.0 protein inference (Step1) parameters tuning.**

A-C. Heatmap reporting the inferred activity (*final score*) of gold standard transcription factors (**A**), kinases/phosphatases (**B**), and other signaling proteins (**C**) using both footprint-based and PhosphoScore methods across 64, 32 and 4 technical conditions (see Supplementary Material).

Precision, Recall, and Root Mean Squared Error (RMSE) are reported for each condition by comparison with the *protein* gold standard (see Methods). The black box highlights the best technical condition set as default in *SignalingProfiler* 2.0 and used in Step 2. Blue and red represent inactive and active proteins, respectively. **D.** For each parameter, the average Precision, Recall, Number of true positives, and Root Mean Squared Error (RMSE) with respect to the proteic gold standard are reported across 64, 32 and 4 conditions for transcription factors and kinases/phosphatases.

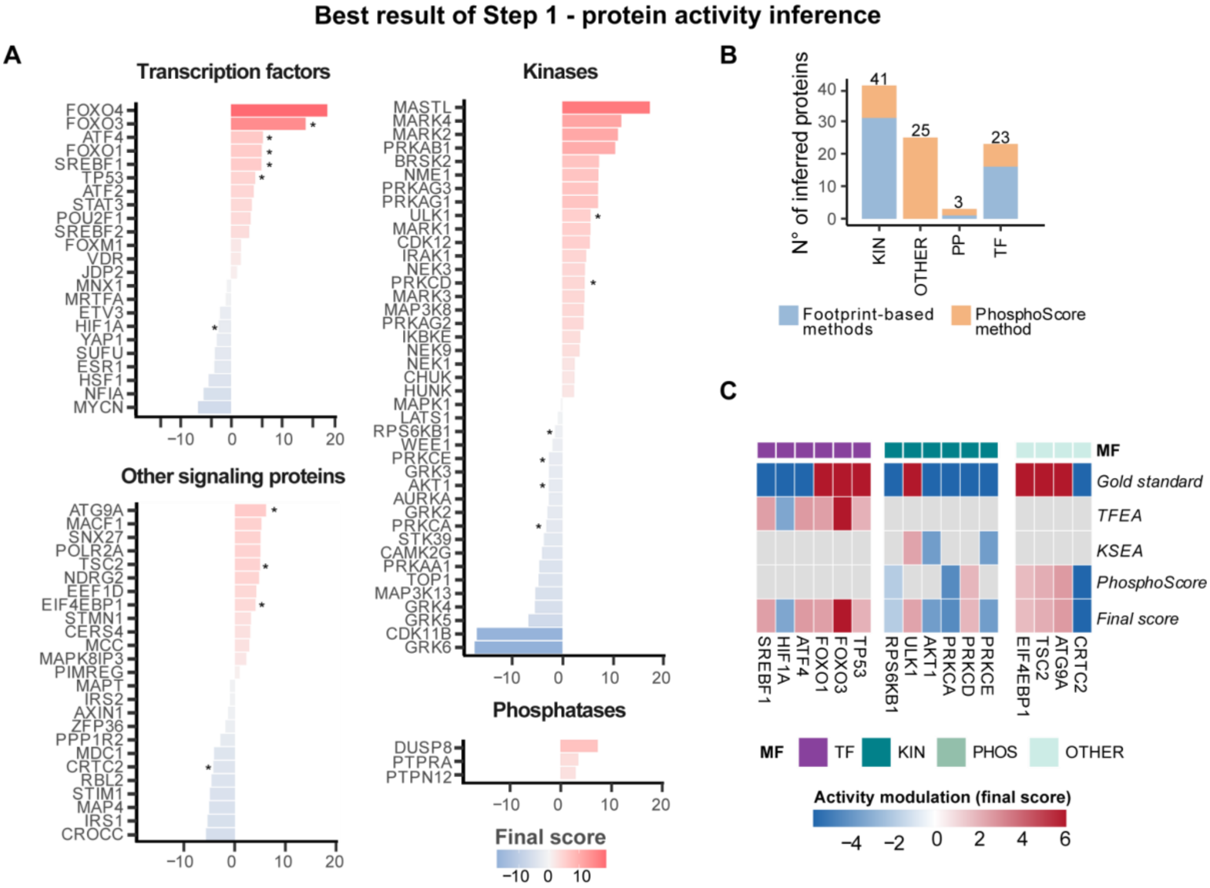

Supplementary Figure 5. The best result of *SignalingProfiler* 2.0 protein inference (Step 1) parameters tuning.

A. Bar plot displaying the activity modulation (metformin-treated vs control condition) for transcription factors, kinases, phosphatases, and other signaling proteins in the top result from Step 1. Blue and red represent inactive and active proteins, respectively.

B. Bar plot representing the proportion of transcription factors, kinases, phosphatases, and other signaling proteins identified using PhosphoScore (orange) or footprint-based methods (blue). **C.** Heatmap summarizing the comparison between the expected activity ("Gold standard") and the activity score in the best technical condition of Step 1.

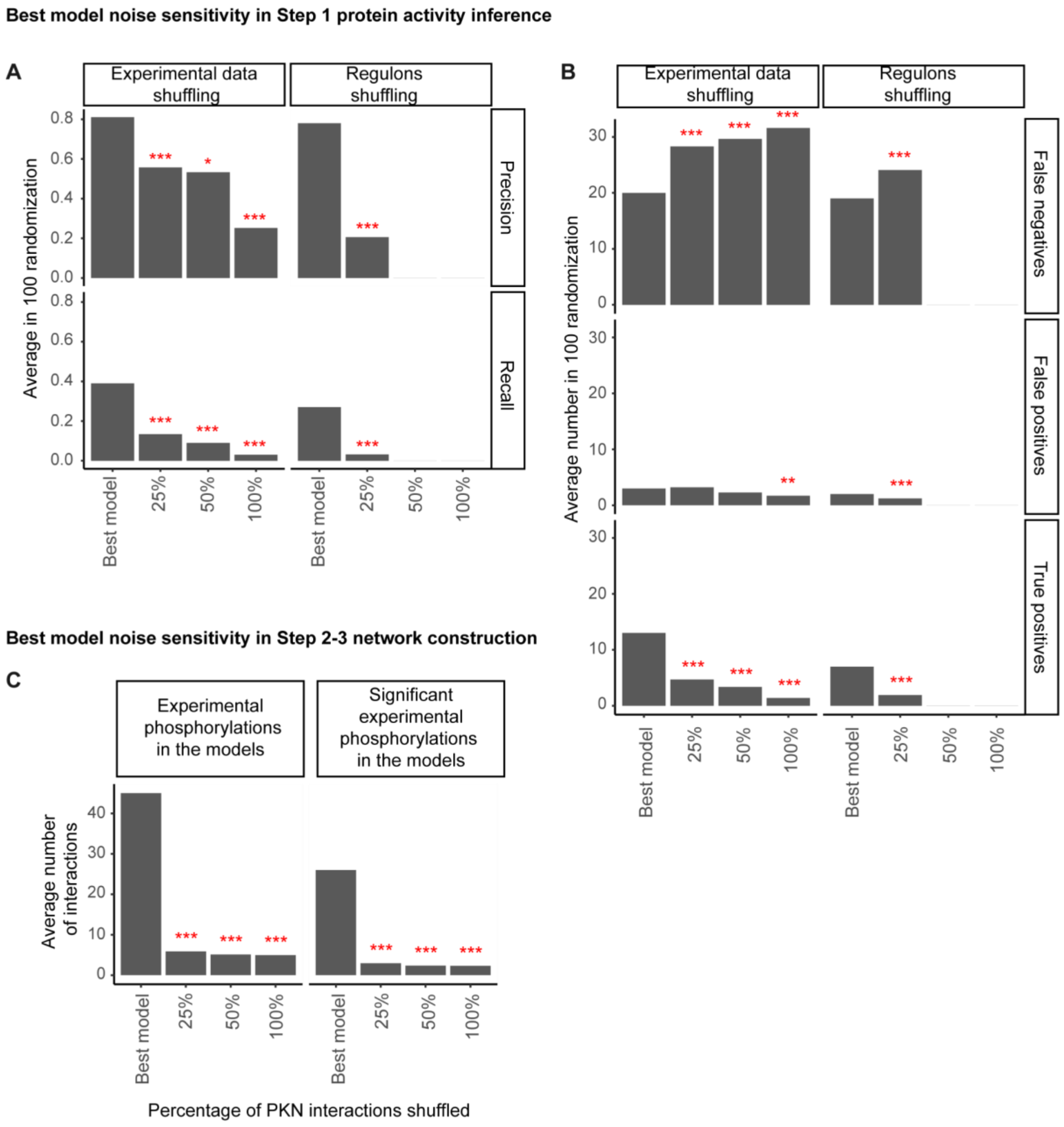

Supplementary Figure 6. ***SignalingProfiler*** **2.0 noise sensitivity analysis.**

A-B. Bar plot reporting the precision, recall, false positives, false negatives, and true positives (y-axis) between inferred proteins resulting from randomized experimental data (**A**) or from randomized regulons (**B**) and the best benchmarking prediction (x-axis). The metrics were evaluated with respect to the protein gold standard. **C.** Bar plot reporting the average number of interactions representing quantified (left panel) or significant (right panel) phosphorylation events in models resulting from randomized prior knowledge networks (PKNs) and best model (x-axis). Percentage of randomized data (25%, 50%, and 100%) is reported. Statistical significance was computed with T-test statistical analysis (p-value < 0.05 *, < 0.01 **, < 0.001 ***).

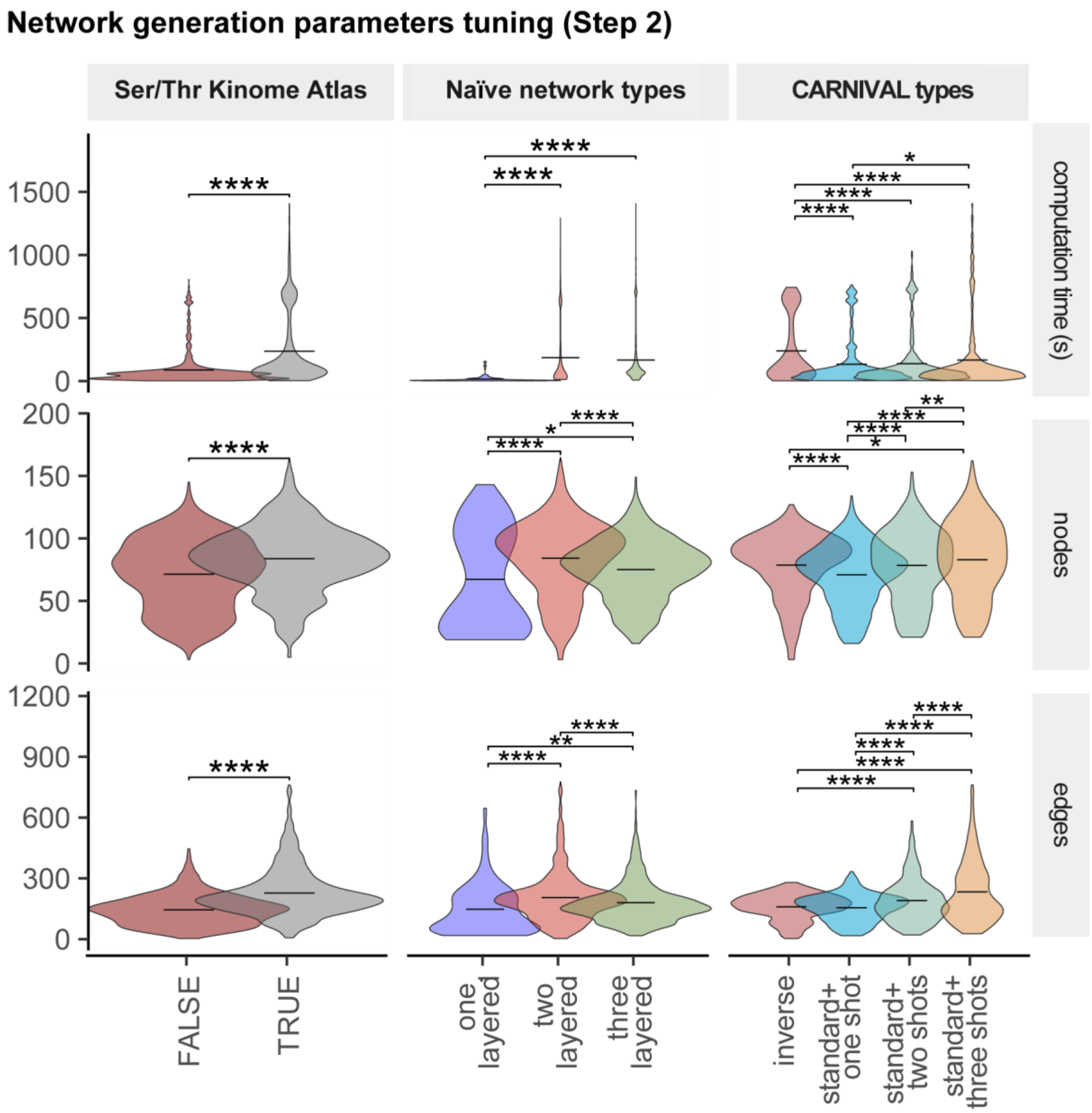

Supplementary Figure 7. ***SignalingProfiler*** **2.0 network generation (Step2) parameters tuning.**

Violin plots reporting the impact of Ser/Thr Kinome Atlas integration in the prior knowledge network, number of layers in the naïve network, and CARNIVAL flavors and types on computation time (in seconds), number of nodes, and edges, across 2989 generated models. The black line indicates the average y value. Statistical significance was computed with T-test statistical analysis (p-value < 0.05 *, < 0.01 **, < 0.001 ***, < 0.0001 ****).

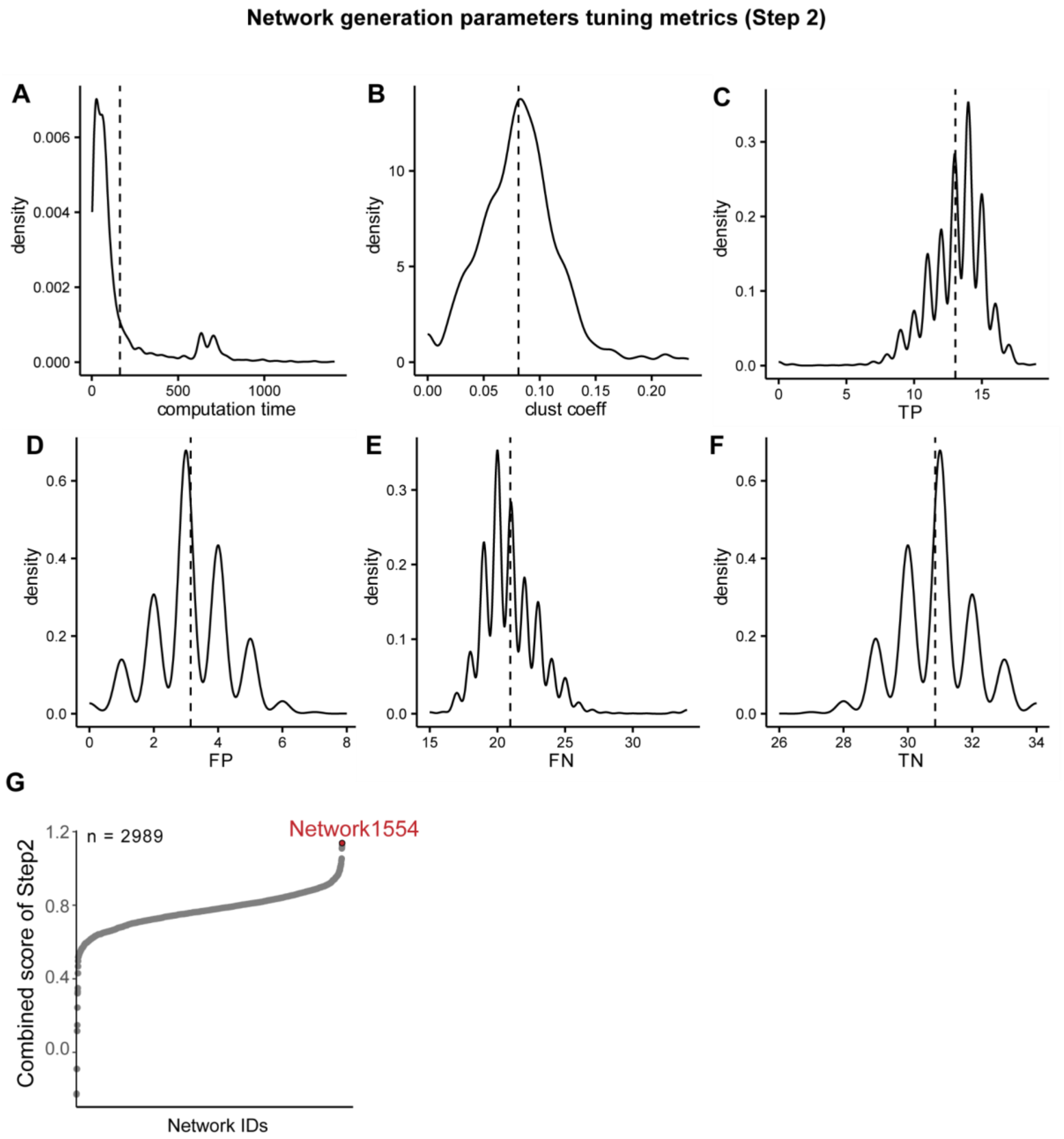

Supplementary Figure 8. *SignalingProfiler* 2.0 network generation (Step 2) tuning metrics. Distribution of different metrics across the family of 2989 generated models (Step 2) **(A-F):** computation time (**A**), number of components (**B**), True Positives (**C**), False Positives (**D**), False Negatives (**E**), True Negatives (**F**) with respect to the gold standard. **G.** Combined score of Step 2 exploited to rank the models.

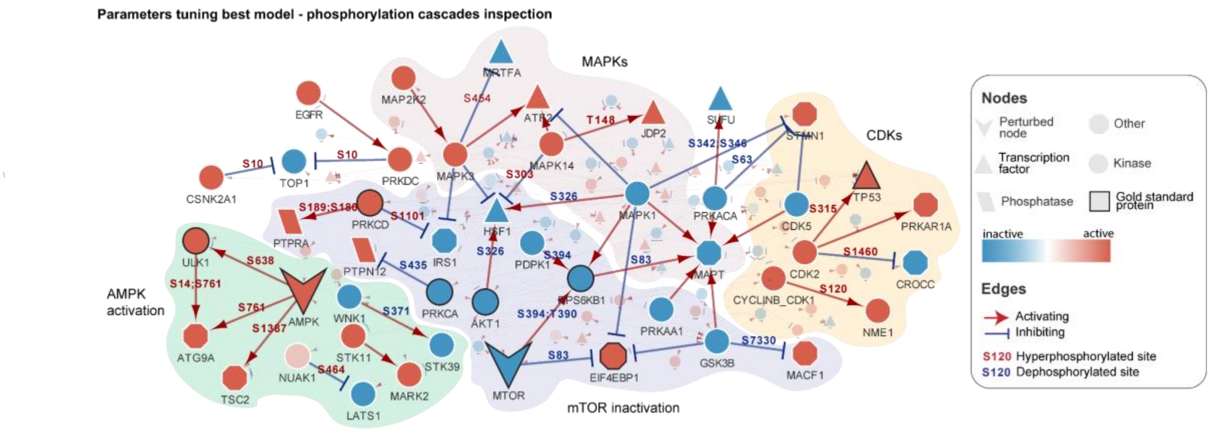

Supplementary Figure 9. The best result of *SignalingProfiler* 2.0 network reconstruction (Step 2) parameters tuning. Visualization of the best model (**Supplementary Figure 8**) of the Step 2 parameters' tuning focused on proteins involved in phosphorylation events significantly deregulated between metformin and control. Nodes and edges are displayed according to the legend. Blue and red nodes represent inactive and active proteins, respectively. Background areas highlight subnetworks associated with known signaling pathways.

Metformin *SignalingProfiler* 2.0 resulting network (109 nodes and 309 edges)

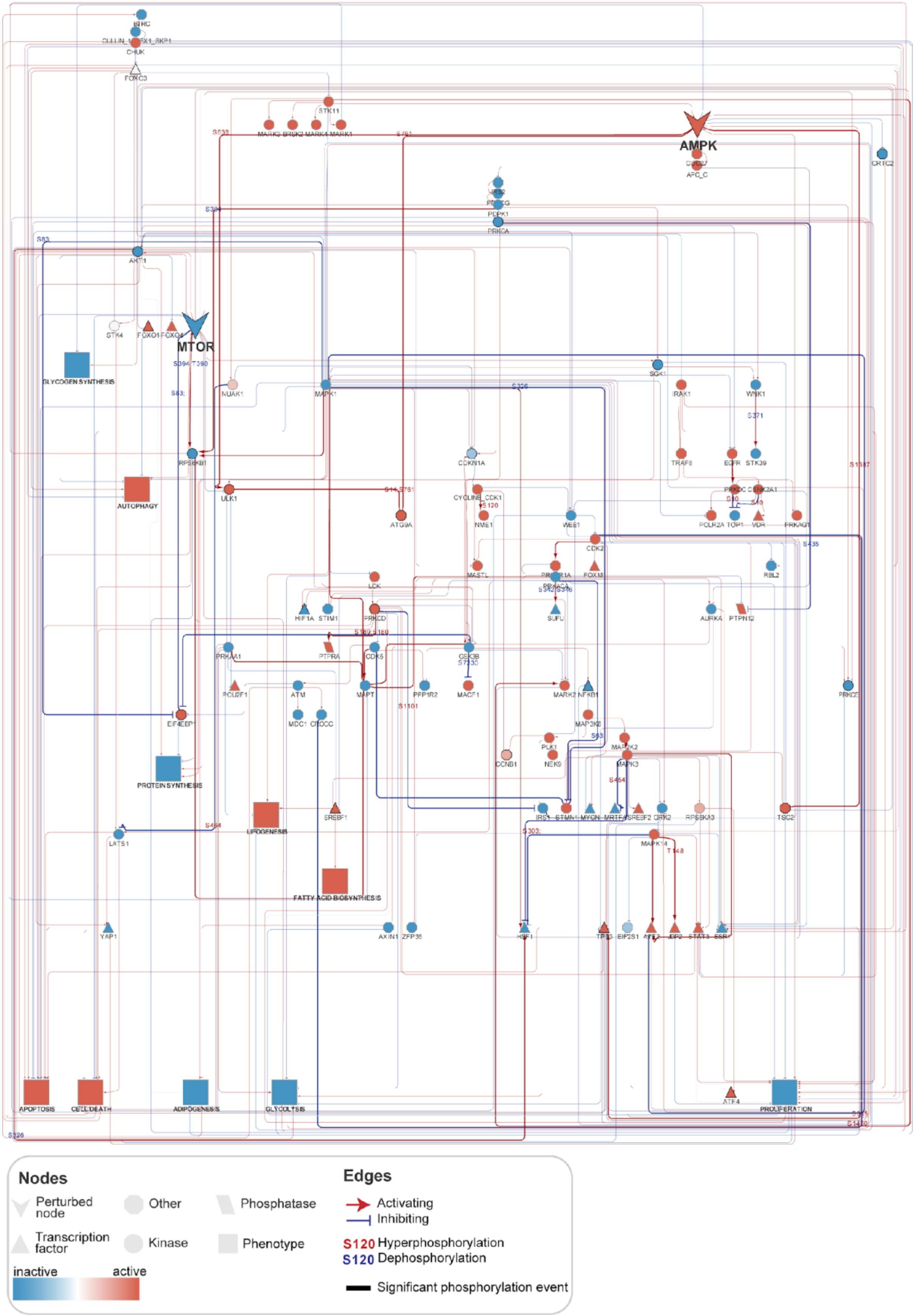

Supplementary Figure 10. The best result of the benchmarking process of *SignalingProfiler* 2.0. Causal network of 109 nodes and 309 edges representing the metformin-induced signaling rewiring in breast cancer cells, starting from AMPK and mTOR proteins and ending on 10 relevant phenotypic traits. Activated and inhibited proteins after metformin treatment, along with activating and inhibiting edges, are indicated in red and blue, respectively. Node shape represents the molecular function. Phosphosites that are mapped on the interactions representing phosphorylation events are colored according to their level of phosphorylation after metformin treatment. Thicker edges represent (de)phosphorylations occurring at phosphosites significantly modulated in experimental data. Browse the model at https://www.ndexbio.org/viewer/networks/fa22e724-b54b-11ee-8a13-005056ae23aa.

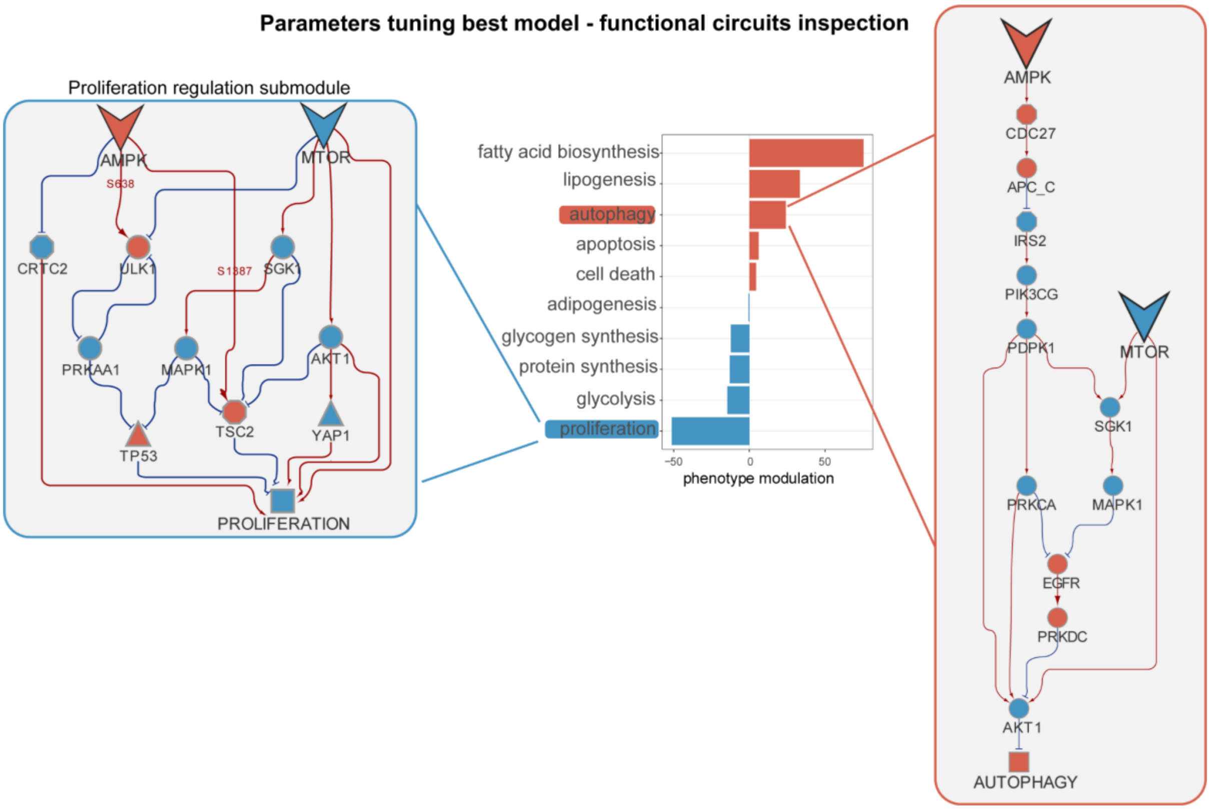

Supplementary Figure 11. The best result of *SignalingProfiler* 2.0 phenotypic inference (Step 3) parameters tuning. Bar plot reporting the inferred modulation of phenotypes upon metformin treatment after Step 3 of *SignalingProfiler* 2.0 (activation in red, inhibition in blue), and the functional circuits extracted from the final model connecting AMPK and mTOR to autophagy and proliferation.

Use case: Massacci et al. (2023)

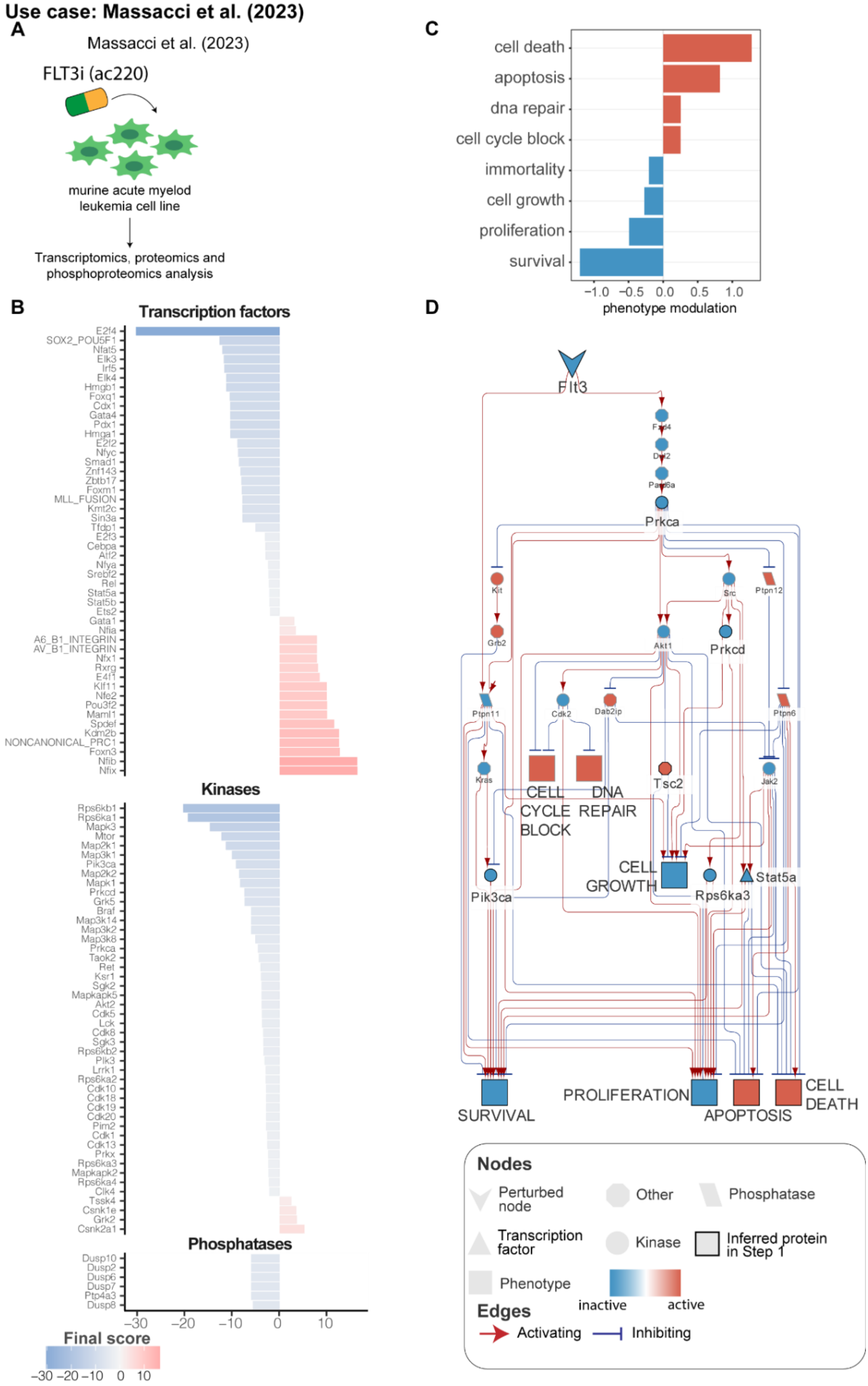

Supplementary Figure 12. Application of *SignalingProfiler* 2.0 to data from acute myeloid leukemia (AML) murine cell line treated with FLT3 kinase inhibitor.

A. Cartoon describing the experimental setup. **B.** Bar plot displaying the activity modulation (FLT3 inhibitor (ac220) vs control condition) for transcription factors, kinases, and phosphatases from multi-omic data from Massacci et al. (2023). Blue and red bars represent inactive and active proteins, respectively. Only proteins with an absolute modulation greater than 2 were represented. **C.** Scatterplot of the protein activity modulation by *SignalingProfiler* 2.0 (x-axis) and *SignalingProfiler* prototype (y-axis). **D.** Bar plot of the phenotypic modulation upon FLT3 inhibitor (ac220) treatment inferred by *SignalingProfiler* 2.0. Blue and red bars represent inactive and active phenotypes, respectively. **E.** Functional circuit impacting inferred phenotypes extracted from the AML model.

Supplementary Data Description

Supplementary Data 1.

Training dataset (Sacco et al., 2016) protein and phenotypic gold standard table.

Supplementary Data 2.

Parameters combinations explanation for *SignalingProfiler* 2.0 step.

Supplementary Data 3.

Inferred proteins list from training dataset in each technical condition of proteins' activity inference (Step 1) parameters tuning.

Supplementary Data 4.

Protein activity inference (Step 1) parameters combinations with quality metrics.

Supplementary Data 5.

Training dataset best result of proteins' activity inference (Step 1) parameters tuning.

Supplementary Data 6.

Network construction (Step 2) parameters combinations with metrics.

Supplementary Data 7.

Phenotypes' activity (Step 3) parameters combinations with metrics.

Supplementary Data 8.

Training dataset model of metformin-induced signaling rewiring returned by the optimal parameters set of *SignalingProfiler* 2.0.

Supplementary Data 9.

Validation datasets (Olsen et al., 2006; Massacci et al., 2023) protein and phenotypic gold standard table.

Supplementary Data 10.

Result of *SignalingProfiler* 2.0 application on Olsen et al. (2006) validation dataset.

Supplementary Data 11.

Result of *SignalingProfiler* 2.0 application on Massacci et al. (2023) validation dataset.

Supplementary Data 12.

Parameters tuning validation results on two validation datasets.

Supplementary Data 13.

Comparison between *SignalingProfiler* prototype and *SignalingProfiler* 2.0.
